# Association of adverse respiratory events with sodium-glucose cotransporter 2 inhibitors versus dipeptidyl peptidase 4 inhibitors among patients with type 2 diabetes in South Korea: a nationwide cohort study

**DOI:** 10.1186/s12916-023-02765-2

**Published:** 2023-02-10

**Authors:** Han Eol Jeong, Sohee Park, Yunha Noh, Sungho Bea, Kristian B. Filion, Oriana H. Y. Yu, Seung Hun Jang, Young Min Cho, Dong Keon Yon, Ju-Young Shin

**Affiliations:** 1 School of Pharmacy, Sungkyunkwan University, Suwon, South Korea; 2 Department of Biohealth Regulatory Science, Sungkyunkwan University, Suwon, South Korea; 3 Departments of Medicine and of Epidemiology, Biostatistics, and Occupational Health, McGill University, Montreal, QC Canada; 4 Centre for Clinical Epidemiology, Lady Davis Institute, Jewish General Hospital, Montreal, QC Canada; 5 Division of Endocrinology and Metabolism, Jewish General Hospital, McGill University, Montreal, QC Canada; 6 Division of Pulmonary, Allergy, and Critical Care Medicine, College of Medicine, Hallym University Sacred Heart Hospital, Hallym University, Anyang, South Korea; 7 Department of Internal Medicine, Seoul National University College of Medicine, Seoul, South Korea; 8 Department of Translational Medicine, Seoul National University College of Medicine, Seoul, South Korea; 9 Department of Internal Medicine, Seoul National University Hospital, Seoul, South Korea; 10 Institute On Aging, Seoul National University, Seoul, South Korea; 11 Medical Science Research Institute, Kyung Hee University College of Medicine, Seoul, South Korea; 12 Department of Pediatrics, Kyung Hee University Medical Center, Kyung Hee University College of Medicine, Seoul, South Korea; 13 Department of Clinical Research Design & Evaluation, Samsung Advanced Institute for Health Sciences & Technology (SAIHST), Sungkyunkwan University, Seoul, South Korea

**Keywords:** Sodium-glucose cotransporter 2 inhibitors, Respiratory effectiveness, Type 2 diabetes, Population-based cohort

## Abstract

**Background:**

Impaired respiratory function remains underrecognized in patients with type 2 diabetes (T2D), despite common pulmonary impairment. Meanwhile, there is little data available on the respiratory effects of sodium glucose cotransporter 2 inhibitors (SGLT2i). Hence, we examined the association between SGLT2i use and the risk of adverse respiratory events in a real-world setting.

**Methods:**

We conducted a population-based, nationwide cohort study using an active-comparator new-user design and nationwide claims data of South Korea from January 2015 to December 2020. Among individuals aged 18 years or older, propensity score matching was done to match each new user of SGLT2is with dipeptidyl peptidase 4 inhibitors (DPP4is), with patients followed up according to an as-treated definition. The primary outcome was respiratory events, a composite endpoint of acute pulmonary edema, acute respiratory distress syndrome (ARDS), pneumonia, and respiratory failure. Secondary outcomes were the individual components of the primary outcome and in-hospital death. Cox models were used to estimate hazard ratios (HRs) and 95% CIs.

**Results:**

Of 205,534 patient pairs in the propensity score matched cohort, the mean age of the entire cohort was 53.8 years and 59% were men, with a median follow-up of 0.66 years; all baseline covariates achieved balance between the two groups. Incidence rates for overall respiratory events were 4.54 and 7.54 per 1000 person-years among SGLT2i and DPP4i users, respectively, corresponding to a rate difference of 3 less events per 1000 person-years (95% CI − 3.44 to − 2.55). HRs (95% CIs) were 0.60 (0.55 to 0.64) for the composite respiratory endpoint, 0.35 (0.23 to 0.55) for acute pulmonary edema, 0.44 (0.18 to 1.05) for ARDS, 0.61 (0.56 to 0.66) for pneumonia, 0.49 (0.31 to 0.76) for respiratory failure, and 0.46 (0.41 to 0.51) for in-hospital death. Similar trends were found across individual SGLT2is, subgroup analyses of age, sex, history of comorbidities, and a range of sensitivity analyses.

**Conclusions:**

These findings suggest a lower risk of adverse respiratory events associated with patients with T2D initiating SGLT2is versus DPP4is. This real-world evidence helps inform patients, clinicians, and guideline writers regarding the respiratory effects of SGLT2i in routine practice.

**Supplementary Information:**

The online version contains supplementary material available at 10.1186/s12916-023-02765-2.

## Background

Type 2 diabetes (T2D) and respiratory disease share a similar pathophysiology background [1, 2]. Impaired respiratory function however remain underrecognized in patients with T2D despite common pulmonary impairment, especially in those with poor glycemic control [3–5]. In particular, glucose levels of the airway surface liquid (ASL), a thin fluid layer at the lung epithelium involved in airway glucose homeostasis, influence respiratory dysfunction [6]; low glucose levels of the ASL help prevent pulmonary infections and the exacerbation of lung diseases [7, 8]. Although antidiabetic drugs reduce glucose levels to restore airway glucose homeostasis [8], accumulating evidence suggests that sodium-glucose cotransporter 2 inhibitors (SGLT2is) may offer greater benefits with respect to respiratory outcomes through various biological mechanisms, such as lowering the ASL glucose levels [9] and reducing airway hyperresponsiveness [10, 11].

Recent clinical interest regarding SGLT2is has largely focused on its cardiorenal benefits, with considerable evidence provided from both randomized trials and observational studies [12–20]. In contrast, only a small number of studies have investigated the respiratory effects of SGLT2is. Meta-analyses of randomized controlled trials showed that SGLT2is reduce the risk of adverse respiratory events versus placebo, including the risks of respiratory disorders [21], pneumonia [22], and respiratory failure [23], as well as the risk of asthma versus dipeptidyl peptidase-4 inhibitors (DPP4is) [24]. However, the respiratory effects of SGLT2is compared with other second or third-line antidiabetic drugs remain less well understood, and there is substantial uncertainty regarding the generalizability of trial findings to real-world settings [25]. Meanwhile, a few observational cohort studies found a lower risk of pneumonia-related outcomes with SGLT2is versus DPP4is [26, 27], and a lower risk of severe, but not moderate, exacerbation of chronic obstructive pulmonary disease with SGLT2is versus sulfonylurea [28]. Despite these findings, to our knowledge, the real-world effect of SGLT2is on non-chronic respiratory outcomes such as acute respiratory distress syndrome (ARDS) or respiratory failure remains unknown in routine practice.

Thus, we aimed to determine whether the use of SGLT2is, compared with the use of DPP4is, is associated with the risk of adverse respiratory events among patients with T2D using a nationwide population-based data from South Korea.

## Methods

### Data source

We conducted a retrospective cohort study using data from the Health Insurance Review and Assessment Service (HIRA) database of South Korea (data number: M20210607316). The HIRA database includes nationwide health insurance claims data on reimbursed healthcare utilization information, including but not limited to diagnoses (International Classification of Diseases, 10^th^ Revision [ICD-10] diagnostic codes, physician’s specialty), procedures (domestic codes), and prescriptions (domestic codes based on the active ingredient, date of prescription, days’ supply, dose) from all settings (inpatient, emergency department, outpatient, and nursing home) [29]. As South Korea has a universal, single-payer health insurance system that is operated and managed exclusively by the National Health Insurance, all residents, domestic or foreign, are registered in the HIRA database [30]. While the HIRA database has information on in-hospital deaths only, 75.6% of all deaths in 2020 were from medical institutions, with 15.6% from homes and 8.8% from other places [31]. The “end of enrollment” date is however not available in the HIRA database, which could prolong the duration of follow-up for non-hospital deaths, although these people would be naturally dis-enrolled from the national claims system. Yet, any misclassified follow-up time due to this issue is likely to be nondifferential between exposure groups to have any impacts on the study.

### Study population

We identified all patients newly prescribed a SGLT2i or DPP4i between 1 Jan 2016 and 31 Dec 2020, given that these medications in this period were indicated and reimbursed exclusively for type 2 diabetes. Inclusion was restricted to new users of SGLT2is or DPP4is, defined as patients prescribed one of these drug classes with no prior record of prescription for either drug class during the prior year. We defined cohort entry as the date of the first prescription for a SGLT2i or a DPP4i. Patients meeting any of the following criteria were excluded: (1) aged less than 18 years on cohort entry to restrict to adults with T2D, (2) initiated both a SGLT2i and a DPP4i on cohort entry to ensure that our exposure groups were mutually exclusive, (3) recorded diagnosis of end-stage renal disease or recipient of dialysis any time before or on the date of cohort entry as these conditions are contraindications for SGLT2is, and (4) recorded diagnosis of any of the respiratory outcomes of interest (acute pulmonary edema, ARDS, pneumonia, and respiratory failure) in the year before or on the date of cohort entry to ensure that outcomes were newly-occurring rather than the sequelae of respiratory outcomes that occurred shortly before cohort entry.

### Exposure

Patients were classified into either the SGLT2i or DPP4i group depending on the drug class prescribed at cohort entry. Exposure was defined using an as-treated approach in which patients were considered exposure to their initial drug from cohort entry until an event or censoring due to switching to or adding the comparator drug, discontinuation of the cohort-entry defining drug, in-hospital death, or end of the study, whichever occurred first. Discontinuation was defined as a treatment gap of at least 30 days between the end of one prescription (defined by the days supply) and the start of the next prescription. We chose DPP4is as the comparator drug to SGLT2is as this drug class has similar indications, formulations, and is used at comparable stages for the treatment of T2D (e.g., second or third-line treatments), with no previous known associations with respiratory events.

### Outcome

The primary outcome of interest was respiratory events, defined as a composite endpoint that included acute pulmonary edema, ARDS, pneumonia, and respiratory failure (Additional file 1: Table S1 for ICD-10 diagnostic codes); these endpoints were selected based on acute, serious events related to abnormal lung function [32–34]. Secondary outcomes were the individual components of the composite respiratory outcome and in-hospital death. Study outcomes, or respiratory events, were defined by the presence of a relevant primary or secondary diagnostic code in an inpatient setting, and the event date was defined by the date of diagnosis. For the primary outcome, the event date was defined as the date of the first occurrence of any component of the composite endpoint. The overall positive predictive value for diagnostic codes from insurance claims was reported to be 82% overall, and 85% from tertiary hospitals for all general conditions, when compared to those from electronic medical records [35].

### Potential confounders and propensity score matching

We assessed sociodemographic characteristics on the date of cohort entry and clinical covariates of comorbidities, co-medication use, level of antidiabetic treatment (proxy for diabetes severity), and healthcare utilization during the year before cohort entry. We then used logistic regression to estimate a propensity score (PS) [36] for the probability of receiving an SGLT2i versus DPP-4, with potential confounders included as independent variables (Additional file 1: Table S2). Level of antidiabetic treatment was categorized into three levels based on the type and number of different antidiabetic drugs classes prescribed in the year prior to cohort entry: level 1, patients not prescribed an antidiabetic drug or treated with only one antidiabetic drug; level 2, patients treated with ≥ 2 different classes of non-insulin antidiabetic drugs; level 3, patients treated with ≥ 1 insulin either alone or in combination with other antidiabetic drugs. SGLT2i and DPP4i users were matched 1:1 using the estimated PS and the nearest neighbor matching algorithm without replacement with a caliper width of 0.05 on the PS scale.

### Statistical analysis

Baseline characteristics were summarized using descriptive statistics, with frequencies and percentages for categorical variables and means and standard deviations (SD) for continuous variables. Potential imbalances in patients’ characteristics were assessed before and after PS matching by using the absolute standardized mean difference (aSMD); aSMD > 0.1 was considered as a meaningful imbalance. Incidence rates of study outcomes per 1000 person-years and the rate differences with 95% confidence interval (CIs) between treatment groups were estimated assuming a Poisson distribution. For the primary analysis, we used cause-specific Cox proportional hazards models within the PS-matched cohort to estimate hazard ratios (HRs) with 95% CIs for the study outcomes associated with SGLT2is versus DPP4is, with in-hospital death treated as a competing event (individuals censored when this competing event occurred). We also evaluated the proportional hazards assumptions using Schoenfeld residuals, which revealed no violations.

### Secondary analyses

In secondary analyses, we subclassified SGLT2i users into four subgroups (dapagliflozin, empagliflozin, ertugliflozin, and ipragliflozin) and compared each individual SGLT2i to DPP4i to estimate the relative effect of each SGLT2i on respiratory events. We also conducted stratified analyses according to age (< 65 and ≥ 65 years), sex, history of chronic obstructive pulmonary disease (COPD), history of asthma, baseline insulin use, and duration of follow-up (< 1 and ≥ 1 year) to assess any effect modifications of these factors on the comparisons between SGLT2is and DPP4is using the Wald test for heterogeneity. We further stratified by the history of cardiovascular disease (CVD), history of heart failure, and the history of chronic kidney disease (CKD) to examine whether the risk of adverse respiratory events associated with SGLT2is differed across these categories.

### Sensitivity analyses

To examine the robustness of our main findings, we performed several sensitivity analyses. First, we repeated the main analysis using an intention-to-treat approach to define follow-up. In this setting, the initial exposure status is carried forward and treatment discontinuation and switching during follow-up were disregarded, thus, patients were followed from cohort entry until the earliest of outcome occurrence, in-hospital death, 365^th^ day of follow-up, or end of the study; the maximum duration of follow-up was limited to 365 days to minimize any bias arising from potential exposure misclassification when there is too lengthy follow-up. Second, we used another method for handling competing risk, the Fine–Gray subdistribution hazard model, to estimate HRs, with in-hospital death treated as a competing event [37]. Third, we varied the grace period duration used to define the exposure effect window from 30 days (primary analysis) to 0 and 60 days. Fourth, we applied a stricter definition for new users of SGLT2is or DPP4is by limiting the study cohort to patients with no record of prescription for the study and comparator drugs in the 2 years prior to cohort entry. Fifth, we defined the exposure of interest as use of a SGLT2i as monotherapy or in combination with metformin. Sixth, to avoid any potential confounding arising from the coronavirus disease 2019 (COVID-19) pandemic or related changes in clinical practice, we shortened the end of the study period from 31 December 2020 to 20 January 2020 (the first positive case of COVID-19 reported in Korea was on 20 January 2020). Seventh, we restricted events to those with primary (or principal) diagnosis codes only to minimize potential outcome misclassification. Eighth, we excluded patients who used insulin during the baseline period to restrict to patients with T2D with similar disease severity. Ninth, we conducted a negative (herpes zoster virus reactivation [38]) and a positive control outcome (hospitalization for heart failure [18]) analysis to assess the possibility of residual confounding in our data. Finally, we calculated E-values to assess the strength of association an unmeasured or unaccounted confounder would need to have to disregard the observed treatment-outcome association [39]. All analyses were performed with SAS Enterprise Guide version 7.1 (SAS Institute).

## Results

### Patients characteristics

**Fig. 1:**
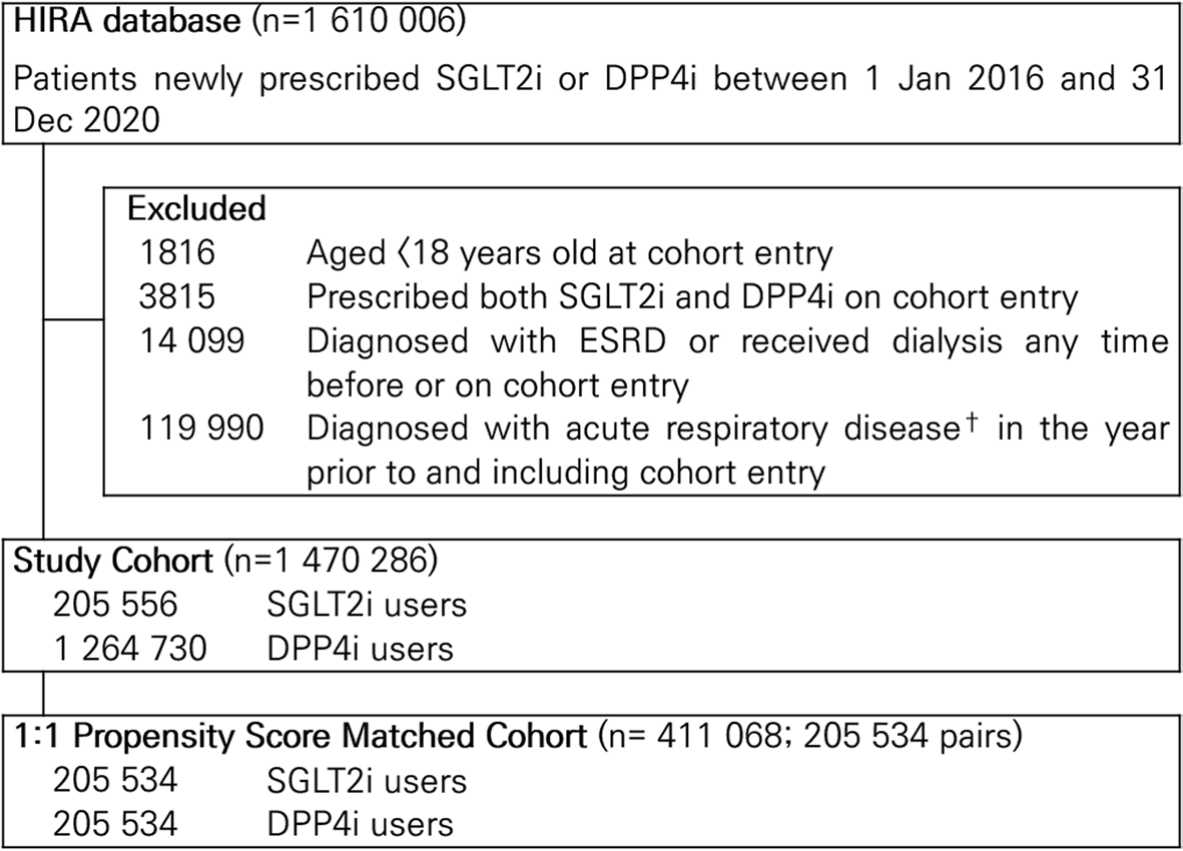
Selection of study and PS-matched cohorts of new users of SGLT2i and DPP4i in South Korea. ^†^Acute respiratory disease includes acute pulmonary edema, acute respiratory syndrome, pneumonia, and respiratory failure. DPP4i, dipeptidyl peptidase-4 inhibitor; ESRD, end-stage renal disease; HIRA, Health Insurance Review and Assessment Service; PS, propensity score; SGLT2i, sodium-glucose cotransporter 2 inhibitors

From 1,610,006 adult patients prescribed SGLT2i and DPP4i, we identified 205,556 users of SGLT2i and 1,264,730 users of DPP4i. After PS matching, 205,534 patients were included in each treatment group; 22 users of a SGLT2i were not matched to users of a DPP4i and thus, were excluded from further analyses (Fig. 1; c-statistic was 0.693 for the PS estimation model). The overall median follow-up duration in the PS-matched cohort was 0.66 years, where the mean duration was 1.14 (SD 1.18) years. All baseline characteristics pre- and post-PS-matching are shown in Table 1. The mean age of the entire PS-matched cohort was 53.8 (SD 13.2) years, and 59.4% were male. All baseline characteristics were well balanced between the treatment groups after PS-matching, with all aSMD < 0.1.

**Table 1:** Baseline characteristics of new users of SGLT2i and users of DPP4i before and after PS matching. Values are numbers (percentages) unless stated otherwise

Characteristics	Pre-matching			Post-matching^a^		
	**DPP4i** **(*n* = 1 264 730)**	**SGLT2i** **(*n* = 205 556)**	**aSMD**	**DPP4i** **(*n* = 205 534)**	**SGLT2i** **(*n* = 205 534)**	**aSMD**
**Follow-up (days; median, IQR)**	311 (98–826)	234 (88–587)		248 (89–636)	234 (88–587)	
**Age (years; mean, SD)**	60.9 (13.4)	53.9 (13.0)	0.53	53.7 (13.4)	53.9 (13.0)	0.01
**Age group (years)**			0.49			0.02
18–44	142,944 (11.3)	48,064 (23.4)		49,838 (24.2)	48,043 (23.4)	
45–64	616,266 (48.7)	115,658 (56.3)		113,334 (55.1)	115,657 (56.3)	
65 ≤	505,520 (40.0)	41,834 (20.4)		42,362 (20.6)	41,834 (20.4)	
**Women**	528,127 (41.8)	83,729 (40.7)	0.02	82,980 (40.4)	83,719 (40.7)	0.01
**Cohort entry year**			0.36			0.01
2016	342,916 (27.1)	32,907 (16.0)		32,459 (15.8)	32,906 (16.0)	
2017	277,909 (22.0)	38,251 (18.6)		38,154 (18.6)	38,249 (18.6)	
2018	236,924 (18.7)	36,777 (17.9)		36,544 (17.8)	36,776 (17.9)	
2019	216,164 (17.1)	48,772 (23.7)		49,169 (23.9)	48,764 (23.7)	
2020	190,817 (15.1)	48,772 (23.7)		49,208 (23.9)	48,764 (23.7)	
**Use of antidiabetic drugs**^**b**^						
Insulin	126,419 (10.0)	19,874 (9.7)	0.01	18,001 (8.8)	19,859 (9.7)	0.03
α-glucosidase inhibitors	45,452 (3.6)	4627 (2.3)	0.08	4287 (2.1)	4626 (2.3)	0.01
GLP-1 receptor agonists	1395 (0.1)	938 (0.5)	0.07	692 (0.3)	919 (0.4)	0.02
Meglitinides	7605 (0.6)	787 (0.4)	0.03	712 (0.3)	786 (0.4)	0.01
Metformin	699,000 (55.3)	106,435 (51.8)	0.07	104,401 (50.8)	106,414 (51.8)	0.02
Sulfonylureas	373,845 (29.6)	46,668 (22.7)	0.16	45,496 (22.1)	46,661 (22.7)	0.01
Thiazolidinediones	67,351 (5.3)	13,711 (6.7)	0.06	12,906 (6.3)	13,705 (6.7)	0.02
**Comorbidities**^**b**^						
Chronic pulmonary disease	124,841 (9.9)	17,601 (8.6)	0.05	16,380 (8.0)	17,601 (8.6)	0.02
Chronic airway disease	121,788 (9.6)	17,258 (8.4)	0.04	16,076 (7.8)	17,258 (8.4)	0.02
Interstitial lung disease	1793 (0.1)	199 (0.1)	0.01	156 (0.1)	199 (0.1)	0.01
Bronchiectasis	4229 (0.3)	459 (0.2)	0.02	426 (0.2)	459 (0.2)	0.00
Cardiovascular disease	638,863 (50.5)	99,229 (48.3)	0.05	96,583 (47.0)	99,215 (48.3)	0.03
Cerebrovascular disease	103,706 (8.2)	10,882 (5.3)	0.12	10,015 (4.9)	10,881 (5.3)	0.02
Cancer^d^	147,799 (11.7)	21,453 (10.4)	0.04	20,084 (9.8)	21,449 (10.4)	0.02
Chronic liver disease	188,315 (14.9)	35,396 (17.2)	0.06	34,347 (16.7)	35,387 (17.2)	0.01
Chronic kidney disease	61,229 (4.8)	8276 (4.0)	0.04	7410 (3.6)	8273 (4.0)	0.02
Diabetic neuropathy	136,349 (10.8)	18,044 (8.8)	0.07	16,638 (8.1)	18,044 (8.8)	0.03
Diabetic retinopathy	177,954 (14.1)	25,645 (12.5)	0.05	24,141 (11.7)	25,639 (12.5)	0.02
Dyslipidemia	452,123 (35.7)	82,537 (40.2)	0.09	81,472 (39.6)	82,524 (40.2)	0.01
Heart failure	21,582 (1.7)	3415 (1.7)	0.01	2707 (1.3)	3415 (1.7)	0.02
Hypoglycemia	6639 (0.5)	566 (0.3)	0.04	506 (0.2)	566 (0.3)	0.01
Obesity	877 (0.1)	545 (0.3)	0.05	263 (0.1)	544 (0.3)	0.03
Obstructive sleep apnea syndrome	2109 (0.2)	880 (0.4)	0.05	481 (0.2)	879 (0.4)	0.03
**Comedications**^**b**^						
ARBs	509,140 (40.3)	84,349 (41.0)	0.02	82,724 (40.2)	84,333 (41.0)	0.02
ACE inhibitors	22,943 (1.8)	3988 (1.9)	0.01	3596 (1.7)	3987 (1.9)	0.01
β-blockers	184,096 (14.6)	30,639 (14.9)	0.01	28,394 (13.8)	30,632 (14.9)	0.03
Calcium channel blockers	444,368 (35.1)	67,705 (32.9)	0.05	65,887 (32.1)	67,695 (32.9)	0.02
Diuretics	283,965 (22.5)	40,197 (19.6)	0.07	38,629 (18.8)	40,194 (19.6)	0.02
Immunosuppressive agents	17,740 (1.4)	2191 (1.1)	0.03	1972 (1.0)	2191 (1.1)	0.01
Inhaled therapy for respiratory disease^c^	33,079 (2.6)	4775 (2.3)	0.02	4315 (2.1)	4775 (2.3)	0.02
NSAIDs	759,011 (60.0)	120,281 (58.5)	0.03	119,567 (58.2)	120,268 (58.5)	0.01
Statins	559,521 (44.2)	95,816 (46.6)	0.05	93,162 (45.3)	95 799 (46.6)	0.03
Systemic antibiotics	791,655 (62.6)	129,820 (63.2)	0.01	128,988 (62.8)	129,807 (63.2)	0.01
Systemic corticosteroids	607,243 (48.0)	97,117 (47.2)	0.02	96,063 (46.7)	97,110 (47.2)	0.01
No. of different classes of non-antidiabetic drugs			0.11			0.03
0–1	900,920 (71.2)	155,845 (75.8)		157,277 (76.5)	155,843 (75.8)	
≥ 2	363,810 (28.8)	49,710 (24.2)		48,257 (23.5)	49,691 (24.2)	
**Level of antidiabetic treatment**			0.111			0.034
1	827,359 (65.4)	144,264 (70.2)		146,783 (71.4)	144,263 (70.2)	
2	310,952 (24.6)	41,418 (20.1)		40,750 (19.8)	41,412 (20.1)	
3	126,419 (10.0)	19,874 (9.7)		18,001 (8.8)	19,859 (9.7)	
**Healthcare use**^**b**^						
Inpatient hospitalizations			0.10			0.01
0	979,075 (77.4)	166,106 (80.8)		168,650 (82.1)	166,091 (80.8)	
1–2	249,204 (19.7)	35,920 (17.5)		33,772 (16.4)	35,916 (17.5)	
≥ 3	36,451 (2.9)	3530 (1.7)		3112 (1.5)	3527 (1.7)	
Number of outpatient physician visits			0.15			0.00
0–2	95,543 (7.6)	15,316 (7.5)		16,074 (7.8)	15,316 (7.5)	
3–5	101,167 (8.0)	19,594 (9.5)		20,231 (9.8)	19,593 (9.5)	
≥ 6	1,068,020 (84.4)	170,646 (83.0)		169,229 (82.3)	170,625 (83.0)	

*ACE* angiotensin-converting enzyme, *ARB* angiotensin II receptor blockers, *aSMD* absolute standardized mean difference, *DPP4i* dipeptidyl peptidase-4 inhibitor, *GLP*-1 glucagon-like peptide-1, *IQR* interquartile range, *NSAID* nonsteroidal anti-inflammatory drugs, *SD* standard deviation, *SGLT2i* sodium-glucose cotransporter 2 inhibitor

^a^Matched each SGLT2i user to one DPP4i user (1:1) using propensity scores, which was estimated by including all baseline covariates as independent variables

^b^Assessed in the three years before study cohort entry and comedication and healthcare use were assessed in the year before study cohort entry

^c^Inhaled therapy for respiratory disease includes β2 agonist inhalants, anticholinergic inhalants, and glucocorticoid inhalants

^d^Excluding non-melanoma skin cancer

### Risk of respiratory events

**Fig. 2:**
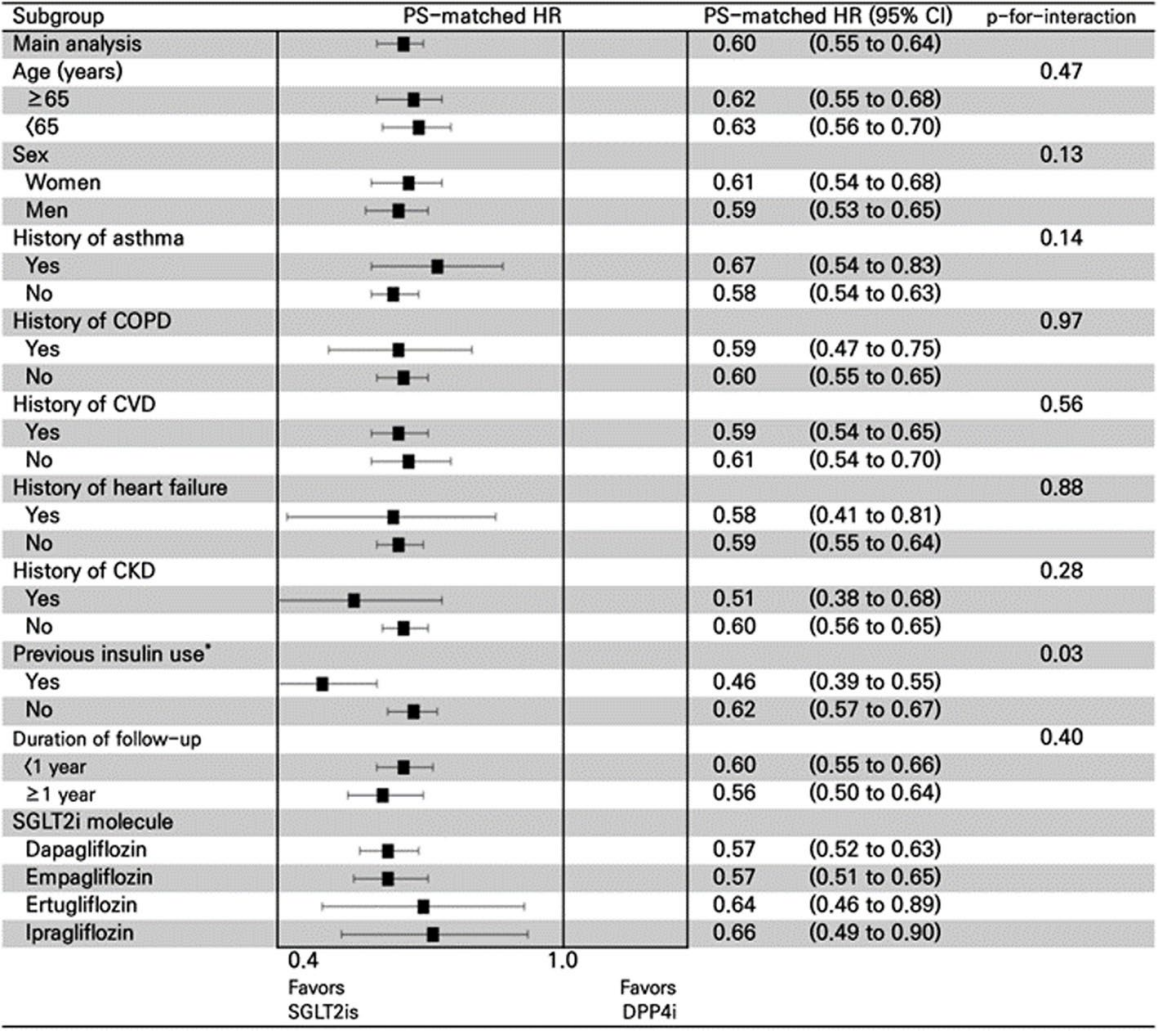
Results for stratified analyses of PS-matched HRs (95% CIs) for adverse respiratory events^**†**^ associated with the use of SGLT2is versus DPP4is. ^†^Defined as a composite end point of acute pulmonary edema, acute respiratory distress syndrome, pneumonia, or respiratory failure. ^*^Prescription for insulin in past year. CI, confidence interval; COPD, chronic obstructive pulmonary disease; CKD, chronic kidney disease; CVD, cardiovascular disease; DPP4i, dipeptidyl peptidase-4 inhibitor; HR, hazard ratio; PS, propensity score; SGLT2i, sodium-glucose cotransporter 2 inhibitor

We found lower incidence rates of adverse respiratory events among SGLT2i users than among DPP4i users (incidence rate 4.54 versus 7.54 events per 1000 person-years; rate difference: − 3.00, 95% CI − 3.44 to − 2.55) in the PS-matched cohort. Within the PS-matched cohort, SGLT2i users had lower risks of the composite respiratory endpoint (HR 0.60, 95% CI 0.55 to 0.64) as well as the secondary outcomes of acute pulmonary edema (HR 0.35, 95% CI 0.23 to 0.55), pneumonia (HR 0.61, 95% CI 0.56 to 0.66), and respiratory failure (HR 0.49, 95% CI 0.31 to 0.76), than DPP4i users. The analysis for ARDS suggested that SGLT2i use was beneficial, but 95% CIs were wide due to sparse data (HR 0.44, 95% CI 0.18 to 1.05) (Table 2). Use of SGLT2i versus DPP4i was also associated with a reduced risk of in-hospital mortality (HR 0.46, 95% CI 0.41 to 0.51) (Additional file 1: Table S3). The analyses for individual SGLT2is showed a similar trend of a lower risk of the composite respiratory event across individual SGLT2i molecules versus DPP4is (Fig. 2; Additional file 1: Table S4).

**Table 2:** PS-matched HRs for association between SGLT2is versus DPP4is and risk of respiratory events and its components

	**No. of events**	**Person-years**	**Incidence rate per** **1000 person-years**		**Rate difference per** **1000 person-years (95% CI)**		**Hazard ratio**^**a**^ **(95% CI)**	
**Respiratory events**^**b**^								
SGLT2i	1025	225,583	4.54	(4.27 to 4.83)	− 3.00	(− 3.44 to − 2.55)	0.60	(0.55 to 0.64)
DPP4i	1810	240,013	7.54	(7.20 to 7.90)	0.00	(Ref)	1.00	(Ref)
**Acute pulmonary edema**								
SGLT2i	26	226,468	0.11	(0.08 to 0.17)	− 0.21	(− 0.29 to − 0.12)	0.35	(0.23 to 0.55)
DPP4i	78	241,692	0.32	(0.26 to 0.40)	0.00	(Ref)	1.00	(Ref)
**ARDS**								
SGLT2i	7	226,478	0.03	(0.01 to 0.06)	− 0.04	(− 0.08 to 0.00)	0.44	(0.18 to 1.05)
DPP4i	17	241,758	0.07	(0.04 to 0.11)	0.00	(Ref)	1.00	(Ref)
**Pneumonia**								
SGLT2i	984	225,605	4.36	(4.10 to 4.64)	− 2.74	(− 3.18 to − 2.31)	0.61	(0.56 to 0.66)
DPP4i	1706	240,090	7.11	(6.78 to 7.45)	0.00	(Ref)	1.00	(Ref)
**Respiratory failure**								
SGLT2i	28	226,467	0.12	(0.09 to 0.18)	− 0.13	(− 0.21 to − 0.05)	0.49	(0.31 to 0.76)
DPP4i	61	241,730	0.25	(0.20 to 0.32)	0.00	(Ref)	1.00	(Ref)

*ARDS* acute respiratory distress syndrome, *CI* confidence interval, *DPP4i* dipeptidyl peptidase-4 inhibitor, *HR* hazard ratio, *PS* propensity score, *SGLT2i* sodium-glucose cotransporter 2 inhibitor

^a^Users of SGLT2is were propensity-score matched to users of DPP4is in a 1:1 ratio

^b^Defined as a composite end point of acute pulmonary edema, acute respiratory distress syndrome, pneumonia, or respiratory failure

### Results of subgroup and stratified analyses

Similar trends for reduced risk of the composite respiratory endpoint were observed among SGLT2i users in each age, sex, and history of asthma, history of COPD, history of CVD, history of CKD, and history of baseline insulin use subgroup. Greater benefits were observed among patients with previous insulin use (HR 0.46, 95% CI 0.39 to 0.55) than among those without previous insulin use (HR 0.62, 95% CI 0.57 to 0.67) (Fig. 2; Additional file 1: Tables S5-S12 for results of each of the stratified analyses).

### Results of sensitivity analyses

**Fig. 3:**
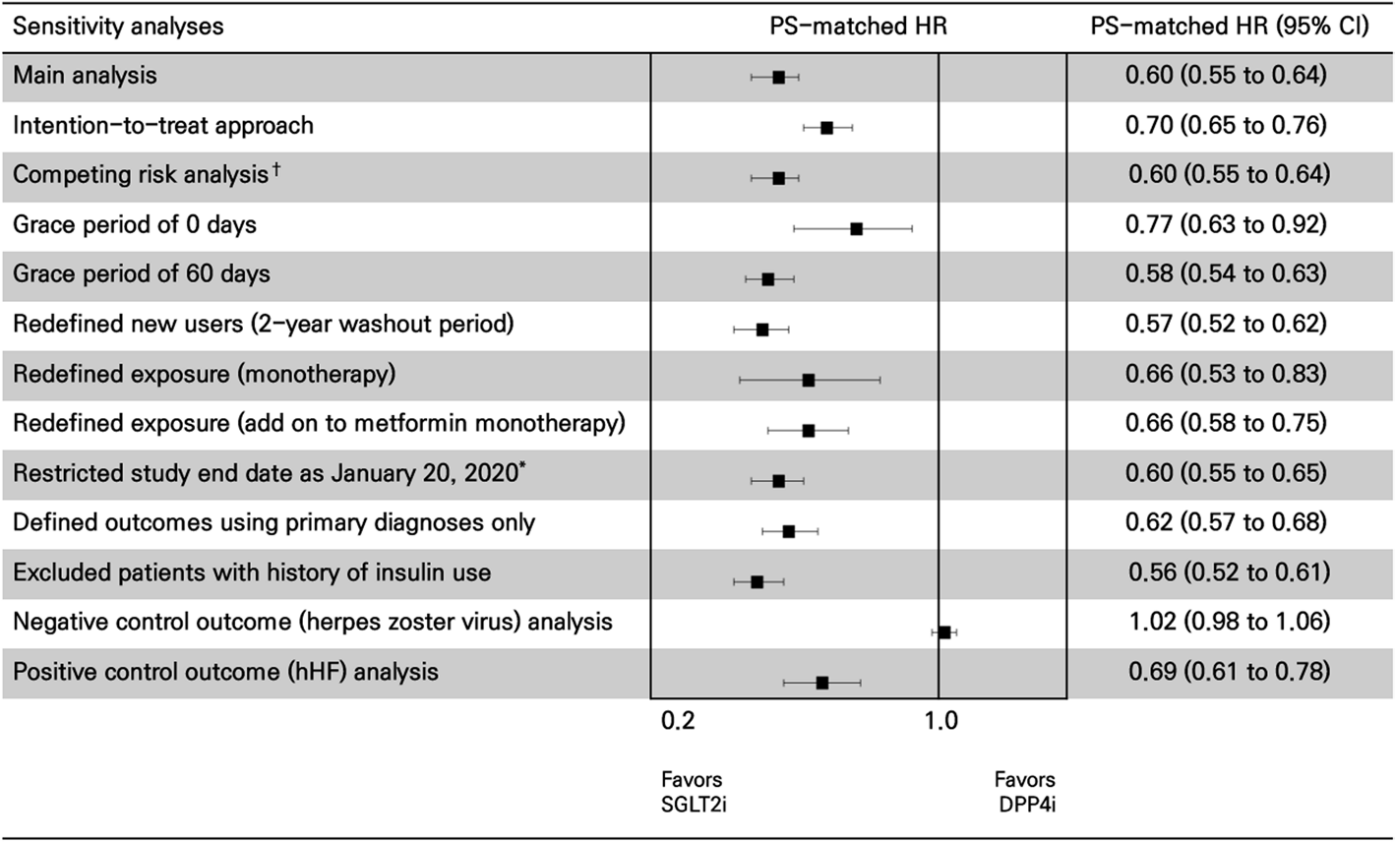
Results for sensitivity analyses of PS-matched HRs (95% CIs) for respiratory events^‡^ associated with the use of SGLT2is versus DPP4is. ^*^First case of COVID-19 reported in South Korea. ^†^Subdistribution hazards with in-hospital death treated as a competing event. ^‡^Defined as a composite end point of acute pulmonary edema, acute respiratory distress syndrome, pneumonia, or respiratory failure. CI, confidence interval; COVID-19, coronavirus disease 2019; DPP4i, dipeptidyl peptidase-4 inhibitors; hHF, hospitalization for heart failure; HR, hazard ratio; PS, propensity score; SGLT2i, sodium-glucose cotransporter 2 inhibitors

Sensitivity analyses that examined study assumptions produced results that were similar to those of our primary analysis (Fig. 3; Additional file 1: Tables S13-22); for instance, the intention-to-treat analysis that did not censor follow-up at treatment interruption or the as-treated analyses that varied the grace period length were consistent to the main analysis (Additional file 1: Table S13). Moreover, the negative control outcome (HR 1.02, 95% CI 0.98 to 1.06) and positive control outcome (HR 0.69, 95% CI 0.61 to 0.78) analyses suggested minimal residual confounding (Additional file 1: Table S15). The *E*-value for point estimates in the as-treated analysis within the PS-matched cohort for respiratory events was 2.72, and 2.50 for CIs; the *E*-values for the secondary outcomes of acute pulmonary edema, ARDS, pneumonia, and respiratory failure were 5.16, 3.97, 2.66, and 3.5 for point estimates, and 3.04, 1.00, 2.40, and 1.96 for CIs, respectively.

## Discussion

### Principal findings

In this nationwide retrospective cohort study, patients with T2D who received a SGLT2i had a 40% lower risk of adverse respiratory events than those receiving a DPP4i in a real-world setting. Similar lower risks were observed for acute pulmonary edema, pneumonia, and respiratory failure. Moreover, similar trends were observed across individual SGLT2i molecules, suggesting a class effect of SGLT2is. Our study findings were supported from a broad range of sensitivity analyses.

### Comparison with other studies

To date, only a few studies have investigated respiratory events associated with SGLT2is, despite its biological plausibility [11]. The lower risk of respiratory events with SGLT2is observed in the present study is consistent with prior meta-analyses that compared adverse respiratory events using data from placebo-controlled randomized trials of SGLT2is; these meta-analyses found a 25% lower risk of overall respiratory disorders [21], a 16% lower risk of pneumonia [22], a 48–60% lower risk of acute pulmonary edema [22, 23], a 29% lower risk of respiratory failure [23], and a 41% lower risk of asthma [24]. Another trial that compared dapagliflozin versus placebo in patients with COVID-19 also hinted at potential respiratory benefits albeit showing an inconclusive lower rate of respiratory decompensation (9.3% versus 11.2%; HR 0.85, 95% CI 0.60 to 1.20) [40]; network meta-analyses in patients with diabetes and COVID-19 also suggested respiratory benefits with SGLT2is by reporting lower COVID-19 mortality [41, 42]. Nevertheless, translating the results of these placebo-controlled trials of SGLT2is with respiratory events to routine clinical practice warrants further studies, given the highly dynamic treatment of T2D in a real-world setting.

No observational study, to our knowledge, has investigated the association between overall respiratory events and SGLT2is using real-world data. We however identified two previous observational cohort studies that examined pneumonia-related outcomes with SGLT2is versus DPP4is by using primary care data from the UK and electronic health records from Hong Kong [26, 27]. Both studies reported a lower risk of pneumonia associated with SGLT2is compared to DPP4is (Hong Kong: HR 0.71, 95% CI 0.62 to 0.81 [27]; United Kingdom: HR 0.48, 95% CI 0.28 to 0.82) [26], which was consistent with our findings (HR 0.60, 95% CI 0.55 to 0.64); subgroup analyses according to the etiology of pneumonia were also consistent (Additional file 1: Tables S23). Moreover, while indirect, our study (HR 59, 95% CI 0.47 to 0.75) was also consistent with a recent cohort study, which used primary care data from the United Kingdom, reporting a lower risk of severe exacerbations of COPD (HR 0.62, 95% CI 0.48 to 0.81) with SGLT2is versus sulfonylureas in patients with T2D and COPD [28]. While few data were available on the real-world, but partial, respiratory effects of SGLT2is, we came across no study, to our knowledge, that made a more comprehensive investigation into several respiratory events simultaneously in this patient population. In the meantime, based on the available evidence from randomized trials and observational studies, our findings provide novel and clinically meaningful real-world evidence by demonstrating that SGLT2is may have respiratory benefits by being associated with a lower risk of non-chronic respiratory events in patients with T2D, given that respiratory mortality, which includes pneumonia, remains a significant public health concern not only in South Korea, but also in Western populations [43].

### Biological plausibility

Pulmonary function deficiency is an underrecognized issue in patients with T2D although these patients have lower lung diffusion capacity than those without T2D, and it has also been associated with an increased risk of hospitalization for pneumonia and subsequent mortality [44, 45]. Consequently, optimal glycemic control in this patient population is essential to improving clinical prognosis, including respiratory disease. While the exact biological mechanism for the favorable effects of SGLT2is on respiratory events remain unknown, several hypotheses exist. Preclinical studies showed that SGLT2is have direct vasodilatory effects on pulmonary circulation that may influence the subsequent risk of pulmonary disease [46, 47]; empagliflozin exhibited pulmonary protective effects after pulmonary ischemia or reperfusion injury in vivo.[48] In addition, SGLT2is facilitate glucose homeostasis of the ASL by reducing glucose movement into the ASL by transcellular pathways via an insulin-independent glucose-lowering mechanism [6, 49]; insulin treatment was previously reported to stimulate cellular glucose uptake [6]. This is important as low ASL glucose concentration helps prevent infections and exacerbation of pulmonary diseases [6]. Furthermore, SGLT2is, via their unique glucose-lowering mechanism that cause energy and salt loss [50], may achieve specific metabolic adaptations and activate aestivation-like hypometabolisms to improve organ’s cellular lifespan [50, 51] to possibly offer cardiorenal benefits [15, 18, 52] and potential hepatic benefits [53–56]. Another possibility that cannot be ruled out is the pleiotropic effects of SGLT2is (e.g., weight and blood pressure control) [57, 58], which could have contributed to lowering the risk of respiratory events in patients with T2D. Although further studies are needed to explore the exact mechanism underlying SGLT2is against respiratory diseases, SGLT2i may indeed have beneficial respiratory effects based on our findings and the existing hypotheses.

### Strengths and limitations of this study

Strengths of this nationwide cohort study is the use of a large-scale real-world data with information on several potential confounders that were used to estimate PS to minimize confounding bias, further accompanied with a robust methodology (e.g., use of an active comparator to minimize confounding by indication bias) and consistent findings in multiple sensitivity analyses. We used DPP4is as an active comparator to SGLT2is because these two antidiabetic treatments are used at a similar stage of T2D treatment and share a common route of administration (e.g., oral regimen). Moreover, we were able to estimate both the class and molecule-specific associations of SGLT2is owing to the large sample size of this study (> 500,000 patients after PS matching).

Nevertheless, some potential limitations exist. First, owing to the inherent limitations of insurance claims data, we could not determine the exact reasons for treatment switches and discontinuations during follow-up (Additional file 1: Tables S24). Furthermore, our findings may be affected by exposure misclassification as we were not able to measure adherence to the medications prescribed. However, results of sensitivity analyses that varied the grace period to define treatment discontinuation in the as-treated analysis or applied the intention-to-treat definition showed consistent findings to that of the main results, implying that any bias arising from exposure misclassification was unlikely to have impacted our findings. Second, while outcome misclassification may have been possible, this is expected to occur non-differentially between the treatment groups and would bias the estimates toward the null hypothesis. To examine this issue, we redefined the study outcomes using only diagnosis codes recorded in the primary position and obtained results that were consistent with those the primary analysis. Third, because our data were claims-based, we did not have information on patients’ lifestyle (e.g., smoking) and detailed laboratory test results (e.g., glycated hemoglobin level). Thus, some residual confounding is possible. However, based on the E-value for our primary outcome, an unmeasured confounder would need to be associated by at least 2.7-fold with both the exposure and outcome to nullify our finding, which we believe is unlikely under reasonable assumptions. Fourth, the median (0.66 years) and mean (1.14 years) duration of follow-up was relatively short to assess the long-term disease-modifying effects of SGLT2is on adverse respiratory events in this study. However, in addition to our mean duration of follow-up being longer than prior studies of SGLT2is [59, 60], similar beneficial effects of SGLT2is were observed in the first year of life as well as in subsequent years in the subgroup analyses that stratified on the duration of follow-up (eTable 17). Future studies using more recent and accumulated real-world data are warranted to examine the long-term respiratory safety of SGLT2is. Fifth, as this study did not investigate into all types of respiratory disease, there are limitations in generalizing the study findings beyond the endpoints studied. Sixth, as an intrinsic limitation of observational studies, our findings only investigated an association of respiratory with SGLT2is versus DPP4is, not its causation. Finally, our study used data from HIRA in South Korea, and more studies using real-world data sources from other regions or countries are needed to confirm the generalizability of our findings, given that analyses examining individual endpoints of the primary composite endpoint, particularly ARDS, produced imprecise estimates.

## Conclusions

In this large, nationwide cohort study, SGLT2i use was associated with reduced risks of overall respiratory events, pneumonia, and respiratory failure compared to DPP4i use among patients with T2D. These respiratory benefits were observed across SGLT2i molecules, suggesting a potential class effect. This real-world evidence helps inform patients, practitioners, and regulatory authorities regarding the respiratory effects of SGLT2i in routine clinical practice.

## Supplementary Information

**Additional file 1: Table S1.** Codes used in cohort selection and outcome definitions. **Table S2.** Covariates included in PS model. **Table S3**. Association between SGLT2is and risk of in-hospital death. **Table S4.** Association between individual SGLT2is and risk of respiratory events. **Table S5**. Age-stratified analysis for the association between SGLT2is and risk of respiratory events. **Table S6**. Sex-stratified analysis for the association between SGLT2is and risk of respiratory events. **Table S7**. Stratified analysis on asthma history for the association between SGLT2is and risk of respiratory events. **Table S8**. Stratified analysis on COPD history for the association between SGLT2is and risk of respiratory events. **Table S9**. Stratified analysis on CVD history for the association between SGLT2is and risk of respiratory events. **Table S10**. Stratified analysis on heart failure history for the association between SGLT2is and risk of respiratory events. **Table S11**. Stratified analysis on CKD history for the association between SGLT2is and risk of respiratory events. **Table S12**. Association between SGLT2is and risk of respiratory events, stratified on follow-up duration. **Table S13.** Sensitivity analysis using an intention-to-treat definition. **Table S14**. Sensitivity analysis that treated in-hospital death as a competing event. **Table S15.** Association between SGLT2is versus DPP4is and risk of negative and positive control outcomes. **Table S16.** Sensitivity analysis that excluded patients with a history of insulin use alone. **Table S17**. Sensitivity analysis that varied the grace period duration. **Table S18**. Sensitivity analysis that used diagnosis codes recorded in the primary position. **Table S19**. Sensitivity analysis that extended the baseline period. **Table S20**. Sensitivity analysis that shortened the end of the study period. **Table S21**. Association between SGLT2is used as monotherapy and risk of respiratory events. **Table S22**. Association between SGLT2is added on to metformin and risk of respiratory events. **Table S23**. Association between SGLT2is and risk of pneumonia, according to its etiology. **Table S24**. Reasons for censoring in the as-treated analysis.

## Data Availability

The data that support the findings of this study are available from the Health Insurance Review and Assessment Service of South Korea but restrictions apply to the availability of these data due to domestic laws and regulations that prohibit the distribution or release of individual’s data to the public, and so are not publicly available. Data are however available from the authors upon reasonable request and with permission of the Health Insurance Review and Assessment Service of South Korea.

## Abbreviations

ARDS: Acute respiratory distress syndrome
ASL: Airway surface liquid
aSMD: Absolute standardized mean difference
CI: Confidence interval
CKD: Chronic kidney disease
COPD: Chronic obstructive pulmonary disease
CVD: Cardiovascular disease
DPP4i: Dipeptidyl peptidase 4 inhibitor
HIRA: Health Insurance Review and Assessment Service
HR: Hazard ratio
ICD-10: International Classification of Diseases, 10th Revision
PS: Propensity score
SGLT2i: Sodium-glucose cotransporter 2 inhibitor
T2D: Type 2 diabetes
