# Supplementary material for: Association of adverse respiratory events with sodium-glucose cotransporter 2 inhibitors versus dipeptidyl peptidase 4 inhibitors among patients with type 2 diabetes in South Korea: a nationwide cohort study
Source: BMC Med. 2023 Feb 10;21:47. doi: 10.1186/s12916-023-02765-2 (PMC9913005; doi:10.1186/s12916-023-02765-2)
Supplement: Supplementary file 1 — Additional file 1: Table S1. Codes used in cohort selection and outcome definitions. Table S2. Covariates included in PS model. Table S3. Association between SGLT2is and risk of in-hospital death. Table S4. Association between individual SGLT2is and risk of respiratory events. Table S5. Age-stratified analysis for the association between SGLT2is and risk of respiratory events. Table S6. Sex-stratified analysis for the association between SGLT2is and risk of respiratory events. Table S7. Stratified analysis on asthma history for the association between SGLT2is and risk of respiratory events. Table S8. Stratified analysis on COPD history for the association between SGLT2is and risk of respiratory events. Table S9. Stratified analysis on CVD history for the association between SGLT2is and risk of respiratory events. Table S10. Stratified analysis on heart failure history for the association between SGLT2is and risk of respiratory events. Table S11. Stratified analysis on CKD history for the association between SGLT2is and risk of respiratory events. Table S12. Association between SGLT2is and risk of respiratory events, stratified on follow-up duration. Table S13. Sensitivity analysis using an intention-to-treat definition. Table S14. Sensitivity analysis that treated in-hospital death as a competing event. Table S15. Association between SGLT2is versus DPP4is and risk of negative and positive control outcomes. Table S16. Sensitivity analysis that excluded patients with a history of insulin use alone. Table S17. Sensitivity analysis that varied the grace period duration. Table S18. Sensitivity analysis that used diagnosis codes recorded in the primary position. Table S19. Sensitivity analysis that extended the baseline period. Table S20. Sensitivity analysis that shortened the end of the study period. Table S21. Association between SGLT2is used as monotherapy and risk of respiratory events. Table S22. Association between SGLT2is added on to metformin and risk of respirat [file 12916_2023_2765_MOESM1_ESM.docx]

**Table S1.** Codes used in cohort selection and outcome definitions.

| **Variable** | **ICD-10, procedure, or ATC code** |
| --- | --- |
| **Eligibility Criteria** | |
| SGLT2i or DPP4i | SGLT2i: A10BK  DPP4i: A10BH |
| End-stage renal disease or dialysis | End-stage renal disease: N185, Z49  Dialysis: O7020, O7061, O7062 |
| History of outcome of interest | Described below |
| **Outcome definition** | |
| Acute pulmonary edema | J81.0 Acute pulmonary edema |
| Acute respiratory distress syndrome | J80.x Acute respiratory distress syndrome |
| Pneumonia | J10.x Influenza due to other identified influenza virus  J11.x Influenza due to unidentified influenza virus  J12.x Viral pneumonia, not elsewhere classified  J13.x Pneumonia due to Streptococcus pneumoniae  J14.x Pneumonia due to Hemophilus influenzae  J15.x Bacterial pneumonia, not elsewhere classified  J16.x Pneumonia due to other infectious organisms, not elsewhere classified  J17.x Pneumonia in diseases classified elsewhere  J18.x Pneumonia, unspecified organism  J22.x Unspecified acute lower respiratory infection  J85.0 Gangrene and necrosis of lung  J85.1 Gangrene and necrosis of lung  J86.x Pyothorax  B01.2 Varicella pneumonia  B05.2 Measles complicated by pneumonia  B25.0 Cytomegaloviral pneumonitis |
| Respiratory failure | J96.x Respiratory failure, not elsewhere classified |

DPP4i, dipeptidyl peptidase 4 inhibitor; ICD-10, International Classification of Diseases, 10th revision; SGLT2i, sodium-glucose cotransporter 2 inhibitor

**Table S2.** Covariates included in PS model

| **Covariates** | **Comment (if applicable)^*^** |
| --- | --- |
| **Demographic** |  |
| Age | Defined at date of cohort entry (reported both as a continuous and categorical variable) |
| Sex |  |
| Health insurance type | National health insurance versus medical aid |
| **Comorbidities** | **ICD-10 codes** |
| Cardiovascular disease | I10.x Essential (primary) hypertension  I11.x Hypertensive heart disease  I12.x Hypertensive chronic kidney disease  I13.x Hypertensive heart and chronic kidney disease  I15.x Secondary hypertension  I20.x Angina pectoris  I21.x Acute myocardial infarction  I22.x Subsequent ST elevation (STEMI) and non-ST elevation (NSTEMI) myocardial infarction  I23.x Certain current complications following STEMI and NSTEMI myocardial infarction (within the 28-day period)  I24.x Other acute ischemic heart diseases  I25.x Chronic ischemic heart disease  I48.x Atrial fibrillation and flutter  I50.x Heart failure  I70.x Atherosclerosis |
| Cerebrovascular disease | I60.x Subarachnoid hemorrhage  I61.x Intracerebral hemorrhage  I62.x Other nontraumatic intracranial hemorrhage  I63.x Cerebral infarction  I64.x Stroke, not specified as hemorrhage or infarction  I65.x Occlusion and stenosis of precerebral arteries, not resulting in cerebral infarction  I66.x Occlusion and stenosis of cerebral arteries, not resulting in cerebral infarction  I67.x Other cerebrovascular diseases  I68.x Cerebrovascular disorders in diseases classified elsewhere  I69.x Sequelae of cerebrovascular disease  G45.x Transient cerebral ischemic attacks and related syndromes  G46.x Vascular syndromes of brain in cerebrovascular diseases |
| Cancer (excluding non-melanoma skin cancer) | C00.x – C14.x Malignant neoplasms of lip, oral cavity and pharynx  C15.x – C26.x Malignant neoplasms of digestive organs  C30.x – C39.x Malignant neoplasms of bone and articular cartilage  C45.x – C49.x Malignant neoplasms of mesothelial and soft tissue  C50.x Malignant neoplasms of breast  C51.x – C58.x Malignant neoplasms of female genital organs  C60.x – C63.x Malignant neoplasms of male genital organs  C64.x – C68.x Malignant neoplasms of urinary tract  C69.x – C72.x Malignant neoplasms of eye, brain and other parts of central nervous system  C73.x – C75.x Malignant neoplasms of thyroid and other endocrine glands  C76.x - C80.x Malignant neoplasms of ill-defined, other secondary and unspecified sites  C81.x - C96.x Malignant neoplasms of lymphoid, hematopoietic and related tissue  D00.x - D09.x In situ neoplasms  D10.x- D36.x Benign neoplasms, except benign neuroendocrine tumors  D37.x - D48.x Neoplasms of uncertain behavior, polycythemia vera and myelodysplastic syndromes |
| Chronic airway disease | J41.x Simple and mucopurulent chronic bronchitis  J42.x Unspecified chronic bronchitis  J43.x Emphysema  J44.x Other chronic obstructive pulmonary disease  J45.x Asthma |
| Interstitial lung disease | J82.x Pulmonary eosinophilia, not elsewhere classified  J84.x Other interstitial pulmonary diseases |
| Bronchiectasis | J47.x Bronchiectasis |
| Chronic liver disease | K70.x Alcoholic liver disease  K71.x Toxic liver disease  K72.x Hepatic failure, not elsewhere classified  K73.x Chronic hepatitis, not elsewhere classified  K74.x Fibrosis and cirrhosis of liver  K75.x Other inflammatory liver diseases  K76.x Other diseases of liver |
| Chronic kidney disease | E10.2 Type 1 diabetes mellitus with kidney complications  E11.2 Type 2 diabetes mellitus with kidney complications  E13.2 Other specified diabetes mellitus with kidney complications  N03.x Chronic nephritic syndrome  N05.x Unspecified nephritic syndrome  N17.x Acute kidney failure  N18.x Chronic kidney disease  N19.x Unspecified kidney failure  N25.x Disorders resulting from impaired renal tubular function  N28.9 Disorder of kidney and ureter, unspecified |
| Diabetic neuropathy | E10.4 Type 1 diabetes mellitus with neurological complications  E11.4 Type 2 diabetes mellitus with neurological complications  E13.4 Other specified diabetes mellitus with neurological complications  G53.x Cranial nerve disorders in diseases classified elsewhere  G56.x Mononeuropathies of upper limb  G57.x Mononeuropathies of lower limb  G58.x Other mononeuropathies  G59.x Mononeuropathy in diseases classified elsewhere  G64.x Other disorders of peripheral nervous system  G62.9 Polyneuropathy, unspecified  G90.0 Idiopathic peripheral autonomic neuropathy  G90.8 Other disorders of autonomic nervous system  G99.0 Autonomic neuropathy in diseases classified elsewhere |
| Diabetic retinopathy | E10.3 Type 1 diabetes mellitus with ophthalmic complications  E11.3 Type 2 diabetes mellitus with ophthalmic complications  E13.3 Other specified diabetes mellitus with ophthalmic complications  H33.x Retinal detachments and breaks  H34.x Retinal vascular occlusions  H35.x Other retinal disorders  H36.x Retinal disorders in diseases classified elsewhere  H54.x Blindness and low vision |
| Dyslipidemia | E78.x Disorders of lipoprotein metabolism and other lipidemia (in the prior 3 years) or C10AA prescription (in the prior year) |
| Heart failure | I50.x Heart failure |
| Hypoglycemia | E16.x Other disorders of pancreatic internal secretion (hospitalization with a diagnosis in any position in the prior 3 years) |
| Obesity | E66.x Overweight and obesity |
| Obstructive sleep apnea syndrome | G47.30 Obstructive sleep apnea |
| **Comedication use** | **ATC codes** |
| ARB | C09C, C09D |
| ACE inhibitors | C09A, C09B |
| β-blockers | C07 |
| Calcium channel blockers | C08, C07FB, C09BB, C09DB |
| Diuretics | C03A, C07B, C07D, C03C, C03E, C03X, C07C, C08G |
| Immunosuppressive agents | L04A |
| Inhaled therapy for respiratory disease^†^ | R03A, R03B |
| Insulin | A10A |
| Meglitinides | A10BX02, A10BX03, A10BX08 |
| Metformin | A10BA02 |
| NSAIDs | M01A |
| Statins | C01AA |
| Sulfonylureas | A10BB |
| Systemic antibiotics | J01 |
| Systemic corticosteroids | H02 |
| Thiazolidinediones | A10BG |
| No. of different classes of non-antidiabetic drugs | Measured by drug class; assessed in the year prior to and including study cohort entry (categorized as 0-1, 2-5, or ≥6) |
| **Level of antidiabetic treatment** | Categorized into three levels based on the type and number of different antidiabetic drugs classes prescribed in the year prior to cohort entry: level 1, patients not prescribed an antidiabetic drug or treated with only one antidiabetic drug; level 2, patients treated with ≥2 different classes of non-insulin antidiabetic drugs; level 3, patients treated with ≥1 insulin either alone or in combination with other antidiabetic drugs. |
| **Healthcare use** |  |
| Number of inpatient hospitalizations | In the year prior to and including study cohort entry (categorized as 0, 1-2, or ≥3) |
| Number of physician visits | Included in- and outpatient visits in the year prior to study cohort entry (categorized as 0-2, 3-5, or ≥6) |

^*^Unless otherwise specified, comorbidities, comedications, and healthcare use were assessed in the year prior to cohort entry. Comorbidities were measured using ICD-10 codes, and procedures were defined using domestic codes.

^†^Inhaled therapy for respiratory disease includes β2 agonist inhalants, anticholinergic inhalants, and glucocorticoid inhalants

ACE, angiotensin-converting enzyme; ARB, angiotensin II receptor blockers; ATC, Anatomical Therapeutic Chemical classification code; ICD-10, International Classification of Diseases, 10th revision; NSAIDs, nonsteroidal anti-inflammatory drugs.

**Table S3** Association between SGLT2is and risk of in-hospital death.

|  | **No of events** | **Person-years** | **Incidence rate per 1000 person-years** | | **Rate difference per 1000 person-years (95% CI)** | | **Hazard ratio^*^ (95% CI)** | |
| --- | --- | --- | --- | --- | --- | --- | --- | --- |
| **In-hospital Death** |  |  |  |  |  |  |  |  |
| SGLT2i (n=206 058) | 457 | 226 479 | 2.02 | (1.84 to 2.21) | -2.36 | (-2.68 to -2.04) | 0.46 | (0.41 to 0.51) |
| DPP4i (n=206 058) | 1058 | 241 761 | 4.38 | (4.12 to 4.65) | 0.00 | (Ref) | 1.00 | (Ref) |
|  |  |  |  |  |  |  |  |  |
| Dapagliflozin (n=123 875) | 252 | 130 868 | 1.93 | (1.70 to 2.18) | -2.44 | (-2.85 to -2.03) | 0.43 | (0.37 to 0.50) |
| DPP4i (n=123 875) | 663 | 151 844 | 4.37 | (4.05 to 4.71) | 0.00 | (Ref) | 1.00 | (Ref) |
|  |  |  |  |  |  |  |  |  |
| Empagliflozin (n=89 959) | 165 | 88 714 | 1.86 | (1.60 to 2.17) | -2.60 | (-3.10 to -2.11) | 0.41 | (0.34 to 0.49) |
| DPP4i (n=89 959) | 460 | 103 046 | 4.46 | (4.07 to 4.89) | 0.00 | (Ref) | 1.00 | (Ref) |
|  |  |  |  |  |  |  |  |  |
| Ertugliflozin (n=11 117) | 13 | 9789 | 1.33 | (0.77 to 2.29) | -3.16 | (-4.41 to -1.90) | 0.27 | (0.15 to 0.49) |
| DDP4i (n=11 117) | 73 | 16 278 | 4.48 | (3.57 to 5.64) | 0.00 | (Ref) | 1.00 | (Ref) |
|  |  |  |  |  |  |  |  |  |
| Ipragliflozin (n=13 889) | 16 | 11 794 | 1.36 | (0.83 to 2.21) | -2.65 | (-3.78 to -1.53) | 0.31 | (0.18 to 0.53) |
| DPP4i (n=13 889) | 75 | 18 704 | 4.01 | (3.20 to 5.03) | 0.00 | (Ref) | 1.00 | (Ref) |

^*^Users of SGLT2is were propensity-score matched to users of DPP4is in a 1:1 ratio.

DPP4i, dipeptidyl peptidase-4 inhibitor; SGLT2i, sodium glucose cotransporter 2 inhibitor

**Table S4** Association between individual SGLT2is and risk of respiratory events.

|  | **No of events** | **Person-years** | **Incidence rate per 1000 person-years** | | **Rate difference per 1000 person-years (95% CI)** | | **Hazard ratio^*^ (95% CI)** | |
| --- | --- | --- | --- | --- | --- | --- | --- | --- |
| **Respiratory events^†^** |  |  |  |  |  |  |  |  |
| Dapagliflozin (n=123 875) | 592 | 130 331 | 4.54 | (4.19 to 4.92) | -3.17 | (-3.75 to -2.60) | 0.57 | (0.52 to 0.63) |
| DPP4i (n=123 875) | 1163 | 150 709 | 7.72 | (7.29 to 8.17) | 0.00 | (Ref) | 1.00 | (Ref) |
|  |  |  |  |  |  |  |  |  |
| Empagliflozin (n=89 959) | 413 | 88 432 | 4.67 | (4.24 to 5.14) | -3.24 | (-3.94 to -2.53) | 0.57 | (0.51 to 0.65) |
| DPP4i (n=89 959) | 809 | 102 324 | 7.91 | (7.38 to 8.47) | 0.00 | (Ref) | 1.00 | (Ref) |
|  |  |  |  |  |  |  |  |  |
| Ertugliflozin (n=11 117) | 52 | 9760 | 5.33 | (4.06 to 6.99) | -2.60 | (-4.59 to -0.60) | 0.64 | (0.46 to 0.89) |
| DDP4i (n=11 117) | 128 | 16 150 | 7.93 | (6.66 to 9.42) | 0.00 | (Ref) | 1.00 | (Ref) |
|  |  |  |  |  |  |  |  |  |
| Ipragliflozin (n=13 889) | 59 | 11 764 | 5.02 | (3.89 to 6.47) | -2.31 | (-4.09 to -0.54) | 0.66 | (0.49 to 0.90) |
| DPP4i (n=13 889) | 136 | 18 562 | 7.33 | (6.19 to 8.67) | 0.00 | (Ref) | 1.00 | (Ref) |
|  |  |  |  |  |  |  |  |  |
| **Acute pulmonary edema** |  |  |  |  |  |  |  |  |
| Dapagliflozin (n=123 875) | 19 | 130 862 | 0.15 | (0.09 to 0.23) | -0.18 | (-0.29 to -0.07) | 0.45 | (0.26 to 0.76) |
| DPP4i (n=123 875) | 49 | 151806 | 0.32 | (0.24 to 0.43) | 0.00 | (Ref) | 1.00 | (Ref) |
|  |  |  |  |  |  |  |  |  |
| Empagliflozin (n=89 959) | 9 | 88 714 | 0.10 | (0.05 to 0.19) | -0.23 | (-0.36 to -0.10) | 0.29 | (0.14 to 0.61) |
| DPP4i (n=89 959) | 34 | 103 021 | 0.33 | (0.24 to 0.46) | 0.00 | (Ref) | 1.00 | (Ref) |
|  |  |  |  |  |  |  |  |  |
| Ertugliflozin (n=11 117) | 2 | 9789 | 0.20 | (0.05 to 0.82) | -0.23 | (-0.65 to 0.20) | 0.57 | (0.12 to 2.82) |
| DDP4i (n=11 117) | 7 | 16 272 | 0.43 | (0.21 to 0.90) | 0.00 | (Ref) | 1.00 | (Ref) |
|  |  |  |  |  |  |  |  |  |
| Ipragliflozin (n=13 889) | 2 | 11 793 | 0.17 | (0.04 to 0.68) | -0.20 | (-0.57 to 0.16) | 0.51 | (0.10 to 2.55) |
| DPP4i (n=13 889) | 7 | 18 697 | 0.37 | (0.18 to 0.79) | 0.00 | (Ref) | 1.00 | (Ref) |
|  |  |  |  |  |  |  |  |  |
| **ARDS** |  |  |  |  |  |  |  |  |
| Dapagliflozin (n=123 875) | 4 | 130 867 | 0.03 | (0.01 to 0.08) | -0.05 | (-0.10 to 0.01) | 0.37 | (0.12 to 1.15) |
| DPP4i (n=123 875) | 12 | 151 841 | 0.08 | (0.04 to 0.14) | 0.00 | (Ref) | 1.00 | (Ref) |
|  |  |  |  |  |  |  |  |  |
| Empagliflozin (n=89 959) | 3 | 88 714 | 0.03 | (0.01 to 0.10) | -0.05 | (-0.12 to 0.02) | 0.37 | (0.10 to 1.36) |
| DPP4i (n=89 959) | 9 | 103 045 | 0.09 | (0.05 to 0.17) | 0.00 | (Ref) | 1.00 | (Ref) |
|  |  |  |  |  |  |  |  |  |
| Ertugliflozin (n=11 117) | 0 | 9789 | 0.00 |  | -0.06 | (-0.18 to 0.06) |  | NA |
| DDP4i (n=11 117) | 1 | 16 278 | 0.06 | (0.01 to 0.44) | 0.00 | (Ref) | 1.00 | (Ref) |
|  |  |  |  |  |  |  |  |  |
| Ipragliflozin (n=13 889) | 0 | 11 794 | 0.00 |  |  | NA |  | NA |
| DPP4i (n=13 889) | 0 | 18 704 | 0.00 |  | 0.00 | (Ref) | 1.00 | (Ref) |
|  |  |  |  |  |  |  |  |  |
| **Pneumonia** |  |  |  |  |  |  |  |  |
| Dapagliflozin (n=123 875) | 565 | 130 346 | 4.33 | (3.99 to 4.71) | -2.96 | (-3.52 to -2.40) | 0.58 | (0.52 to 0.64) |
| DPP4i (n=123 875) | 1100 | 150 756 | 7.30 | (6.88 to 7.74) | 0.00 | (Ref) | 1.00 | (Ref) |
|  |  |  |  |  |  |  |  |  |
| Empagliflozin (n=89 959) | 399 | 88 433 | 4.51 | (4.09 to 4.98) | -2.92 | (-3.61 to -2.23) | 0.59 | (0.52 to 0.67) |
| DPP4i (n=89 959) | 761 | 102 356 | 7.43 | (6.92 to 7.98) | 0.00 | (Ref) | 1.00 | (Ref) |
|  |  |  |  |  |  |  |  |  |
| Ertugliflozin (n=11 117) | 51 | 9760 | 5.23 | (3.97 to 6.88) | -2.20 | (-4.16 to -0.25) | 0.67 | (0.48 to 0.93) |
| DDP4i (n=11 117) | 120 | 16 158 | 7.43 | (6.21 to 8.88) | 0.00 | (Ref) | 1.00 | (Ref) |
|  |  |  |  |  |  |  |  |  |
| Ipragliflozin (n=13 889) | 58 | 11 764 | 4.93 | (3.81 to 6.38) | -1.96 | (-3.70 to -0.22) | 0.69 | (0.59 to 0.94) |
| DPP4i (n=13 889) | 128 | 18 570 | 6.89 | (5.80 to 8.20) | 0.00 | (Ref) | 1.00 | (Ref) |
|  |  |  |  |  |  |  |  |  |
| **Respiratory failure** |  |  |  |  |  |  |  |  |
| Dapagliflozin (n=123 875) | 18 | 130 858 | 0.14 | (0.09 to 0.22) | -0.12 | (-0.22 to -0.02) | 0.53 | (0.31 to 0.94) |
| DPP4i (n=123 875) | 39 | 151 825 | 0.26 | (0.19 to 0.35) | 0.00 | (Ref) | 1.00 | (Ref) |
|  |  |  |  |  |  |  |  |  |
| Empagliflozin (n=89 959) | 9 | 88 712 | 0.10 | (0.05 to 0.19) | -0.28 | (-0.41 to -0.14) | 0.28 | (0.13 to 0.57) |
| DPP4i (n=89 959) | 39 | 103 029 | 0.38 | (0.28 to 0.52) | 0.00 | (Ref) | 1.00 | (Ref) |
|  |  |  |  |  |  |  |  |  |
| Ertugliflozin (n=11 117) | 0 | 9789 | 0.00 |  | -0.18 | (-0.39 to 0.02) |  | NA |
| DDP4i (n=11 117) | 3 | 16 276 | 0.18 | (0.06 to 0.57) | 0.00 | (Ref) | 1.00 | (Ref) |
|  |  |  |  |  |  |  |  |  |
| Ipragliflozin (n=13 889) | 0 | 11 794 | 0.00 |  | -0.32 | (-0.58 to -0.06) |  | NA |
| DPP4i (n=13 889) | 6 | 18 702 | 0.32 | (0.14 to 0.71) | 0.00 | (Ref) | 1.00 | (Ref) |

^*^Users of SGLT2is were propensity-score matched to users of DPP4is in a 1:1 ratio.

^†^Defined as a composite end point of acute pulmonary edema, acute respiratory distress syndrome, pneumonia, or respiratory failure.

ARDS, acute respiratory distress syndrome; DPP4i, dipeptidyl peptidase-4 inhibitor; SGLT2i, sodium glucose cotransporter 2 inhibitor

**Table S5** Age-stratified analysis for the association between SGLT2is and risk of respiratory events.

|  | **No of events** | **Person-years** | **Incidence rate per 1000 person-years** | | **Rate difference per 1000 person-years (95% CI)** | | **Hazard ratio^*^ (95% CI)** | |
| --- | --- | --- | --- | --- | --- | --- | --- | --- |
|  |  |  |  |  |  |  |  |  |
| **Aged <65 years** |  |  |  |  |  |  |  |  |
| **Respiratory events^†^** |  |  |  |  |  |  |  |  |
| SGLT2i (n=163 700) | 550 | 180 515 | 3.05 | (2.80 to 3.31) | -1.86 | (-2.27 to -1.45) | 0.62 | (0.55 to 0.68) |
| DPP4i (n=163 172) | 905 | 184 326 | 4.91 | (4.60 to 5.24) | 0.00 | (Ref) | 1.00 | (Ref) |
| **Acute pulmonary edema** |  |  |  |  |  |  |  |  |
| SGLT2i | 8 | 181 080 | 0.04 | (0.02 to 0.09) | -0.11 | (-0.17 to -0.04) | 0.29 | (0.13 to 0.64) |
| DPP4i | 28 | 185 260 | 0.15 | (0.10 to 0.22) | 0.00 | (Ref) | 1.00 | (Ref) |
| **ARDS** |  |  |  |  |  |  |  |  |
| SGLT2i | 2 | 181 083 | 0.01 | (0.00 to 0.04) | -0.02 | (-0.04 to 0.01) | 0.41 | (0.08 to 2.11) |
| DPP4i | 5 | 185 280 | 0.03 | (0.01 to 0.06) | 0.00 | (Ref) | 1.00 | (Ref) |
| **Pneumonia** |  |  |  |  |  |  |  |  |
| SGLT2i | 534 | 180 527 | 2.96 | (2.72 to 3.22) | -1.73 | (-2.13 to -1.33) | 0.63 | (0.56 to 0.70) |
| DPP4i | 864 | 184 356 | 4.69 | (4.38 to 5.01) | 0.00 | (Ref) | 1.00 | (Ref) |
| **Respiratory failure** |  |  |  |  |  |  |  |  |
| SGLT2i | 13 | 181 074 | 0.07 | (0.04 to 0.12) | -0.07 | (-0.14 to -0.00) | 0.50 | (0.26 to 0.98) |
| DPP4i | 26 | 185 264 | 0.14 | (0.10 to 0.21) | 0.00 | (Ref) | 1.00 | (Ref) |
|  |  |  |  |  |  |  |  |  |
| **Aged ≥65 years** |  |  |  |  |  |  |  |  |
| **Respiratory events^†^** |  |  |  |  |  |  |  |  |
| SGLT2i (n=41 834) | 475 | 45 068 | 10.54 | (9.63 to 11.53) | -5.71 | (-7.13 to -4.29) | 0.63 | (0.56 to 0.70) |
| DPP4i (n=42 362) | 905 | 55 687 | 16.25 | (15.23 to 17.35) | 0.00 | (Ref) | 1.00 | (Ref) |
| **Acute pulmonary edema** |  |  |  |  |  |  |  |  |
| SGLT2i | 18 | 45 387 | 0.40 | (0.25 to 0.63) | -0.49 | (-0.80 to -0.18) | 0.44 | (0.26 to 0.75) |
| DPP4i | 50 | 56 432 | 0.89 | (0.67 to 1.17) | 0.00 | (Ref) | 1.00 | (Ref) |
| **ARDS** |  |  |  |  |  |  |  |  |
| SGLT2i | 5 | 45 395 | 0.11 | (0.05 to 0.26) | -0.10 | (-0.26 to 0.05) | 0.50 | (0.17 to 1.41) |
| DPP4i | 12 | 56 478 | 0.21 | (0.12 to 0.37) | 0.00 | (Ref) | 1.00 | (Ref) |
| **Pneumonia** |  |  |  |  |  |  |  |  |
| SGLT2i | 450 | 45 078 | 9.98 | (9.10 to 10.95) | -5.12 | (-6.50 to -3.75) | 0.64 | (0.57 to 0.72) |
| DPP4i | 842 | 55 734 | 15.11 | (14.12 to 16.16) | 0.00 | (Ref) | 1.00 | (Ref) |
| **Respiratory failure** |  |  |  |  |  |  |  |  |
| SGLT2i | 15 | 45 393 | 0.33 | (0.20 to 0.55) | -0.29 | (-0.55 to -0.02) | 0.52 | (0.28 to 0.96) |
| DPP4i | 35 | 56 466 | 0.62 | (0.45 to 0.86) | 0.00 | (Ref) | 1.00 | (Ref) |

^*^Users of SGLT2is were propensity-score matched to users of DPP4is in a 1:1 ratio.

^†^Defined as a composite end point of acute pulmonary edema, acute respiratory distress syndrome, pneumonia, or respiratory failure.

ARDS, acute respiratory pulmonary syndrome; DPP4i, dipeptidyl peptidase-4 inhibitor; SGLT2i, sodium glucose cotransporter 2 inhibitor

**Table S6** Sex-stratified analysis for the association between SGLT2is and risk of respiratory events.

|  | **No of events** | **Person-years** | **Incidence rate per 1000 person-years** | | **Rate difference per 1000 person-years (95% CI)** | | **Hazard ratio^*^ (95% CI)** | |
| --- | --- | --- | --- | --- | --- | --- | --- | --- |
|  |  |  |  |  |  |  |  |  |
| **Male** |  |  |  |  |  |  |  |  |
| **Respiratory events^†^** |  |  |  |  |  |  |  |  |
| SGLT2i (n=121 815) | 544 | 131 397 | 4.14 | (3.81 to 4.50) | -2.84 | (-3.40 to -2.28) | 0.59 | (0.53 to 0.65) |
| DPP4i (n=122 554) | 968 | 138 700 | 6.98 | (6.55 to 7.43) | 0.00 | (Ref) | 1.00 | (Ref) |
| **Acute pulmonary edema** |  |  |  |  |  |  |  |  |
| SGLT2i | 13 | 131 821 | 0.10 | (0.06 to 0.17) | -0.18 | (-0.28 to -0.08) | 0.36 | (0.19 to 0.67) |
| DPP4i | 39 | 139 509 | 0.28 | (0.20 to 0.38) | 0.00 | (Ref) | 1.00 | (Ref) |
| **ARDS** |  |  |  |  |  |  |  |  |
| SGLT2i | 6 | 131 825 | 0.05 | (0.02 to 0.10) | -0.02 | (-0.07 to 0.04) | 0.71 | (0.25 to 2.00) |
| DPP4i | 9 | 139 551 | 0.06 | (0.03 to 0.12) | 0.00 | (Ref) | 1.00 | (Ref) |
| **Pneumonia** |  |  |  |  |  |  |  |  |
| SGLT2i | 521 | 131 406 | 3.96 | (3.64 to 4.32) | -2.63 | (-3.18 to -2.08) | 0.60 | (0.54 to 0.66) |
| DPP4i | 915 | 138 752 | 6.59 | (6.18 to 7.04) | 0.00 | (Ref) | 1.00 | (Ref) |
| **Respiratory failure** |  |  |  |  |  |  |  |  |
| SGLT2i | 17 | 131 821 | 0.13 | (0.08 to 0.21) | -0.14 | (-0.24 to -0.03) | 0.48 | (0.27 to 0.86) |
| DPP4i | 37 | 139 529 | 0.27 | (0.19 to 0.37) | 0.00 | (Ref) | 1.00 | (Ref) |
|  |  |  |  |  |  |  |  |  |
| **Female** |  |  |  |  |  |  |  |  |
| **Respiratory events^†^** |  |  |  |  |  |  |  |  |
| SGLT2i (n=83 719) | 481 | 94 186 | 5.11 | (4.67 to 5.58) | -3.20 | (-3.93 to -2.48) | 0.61 | (0.54 to 0.68) |
| DPP4i (n=82 980) | 842 | 101 313 | 8.31 | (7.77 to 8.89) | 0.00 | (Ref) | 1.00 | (Ref) |
| **Acute pulmonary edema** |  |  |  |  |  |  |  |  |
| SGLT2i | 13 | 94 647 | 0.14 | (0.08 to 0.24) | -0.24 | (-0.39 to -0.10) | 0.35 | (0.19 to 0.66) |
| DPP4i | 39 | 102 183 | 0.38 | (0.28 to 0.52) | 0.00 | (Ref) | 1.00 | (Ref) |
| **ARDS** |  |  |  |  |  |  |  |  |
| SGLT2i | 1 | 94 652 | 0.01 | (0.00 to 0.08) | -0.07 | (-0.13 to -0.01) | 0.13 | (0.02 to 1.03) |
| DPP4i | 8 | 102 207 | 0.08 | (0.04 to 0.16) | 0.00 | (Ref) | 1.00 | (Ref) |
| **Pneumonia** |  |  |  |  |  |  |  |  |
| SGLT2i | 463 | 94 198 | 4.92 | (4.49 to 5.38) | -2.89 | (-3.59 to -2.19) | 0.62 | (0.55 to 0.70) |
| DPP4i | 791 | 101 338 | 7.81 | (7.28 to 8.37) | 0.00 | (Ref) | 1.00 | (Ref) |
| **Respiratory failure** |  |  |  |  |  |  |  |  |
| SGLT2i | 11 | 94 646 | 0.12 | (0.06 to 0.21) | -0.12 | (-0.23 to -0.00) | 0.49 | (0.24 to 1.00) |
| DPP4i | 24 | 102 202 | 0.23 | (0.16 to 0.35) | 0.00 | (Ref) | 1.00 | (Ref) |

^*^Users of SGLT2is were propensity-score matched to users of DPP4is in a 1:1 ratio.

^†^Defined as a composite end point of acute pulmonary edema, acute respiratory distress syndrome, pneumonia, or respiratory failure.

ARDS, acute respiratory pulmonary syndrome; DPP4i, dipeptidyl peptidase-4 inhibitor; SGLT2i, sodium glucose cotransporter 2 inhibitor

**Table S7** Stratified analysis on asthma history for the association between SGLT2is and risk of respiratory events.

|  | **No of events** | **Person-years** | **Incidence rate per 1000 person-years** | | **Rate difference per 1000 person-years (95% CI)** | | **Hazard ratio^*^ (95% CI)** | |
| --- | --- | --- | --- | --- | --- | --- | --- | --- |
|  |  |  |  |  |  |  |  |  |
| **No History of Asthma** |  |  |  |  |  |  |  |  |
| **Respiratory events^†^** |  |  |  |  |  |  |  |  |
| SGLT2i (n=194 848) | 888 | 213 590 | 4.16 | (3.89 to 4.44) | -2.90 | (-3.34 to -2.46) | 0.58 | (0.54 to 0.63) |
| DPP4i (n=195 816) | 1610 | 228 130 | 7.06 | (6.72 to 7.41) | 0.00 | (Ref) | 1.00 | (Ref) |
| **Acute pulmonary edema** |  |  |  |  |  |  |  |  |
| SGLT2i | 22 | 214 371 | 0.10 | (0.07 to 0.16) | -0.18 | (-0.26 to -0.10) | 0.36 | (0.22 to 0.59) |
| DPP4i | 65 | 229 611 | 0.28 | (0.22 to 0.36) | 0.00 | (Ref) | 1.00 | (Ref) |
| **ARDS** |  |  |  |  |  |  |  |  |
| SGLT2i | 6 | 214 377 | 0.03 | (0.01 to 0.06) | -0.04 | (-0.08 to -0.00) | 0.40 | (0.16 to 1.02) |
| DPP4i | 16 | 229 669 | 0.07 | (0.04 to 0.11) | 0.00 | (Ref) | 1.00 | (Ref) |
| **Pneumonia** |  |  |  |  |  |  |  |  |
| SGLT2i | 855 | 213 606 | 4.00 | (3.74 to 4.28) | -2.66 | (-3.09 to -2.23) | 0.59 | (0.55 to 0.65) |
| DPP4i | 1520 | 228 197 | 6.66 | (6.33 to 7.00) | 0.00 | (Ref) | 1.00 | (Ref) |
| **Respiratory failure** |  |  |  |  |  |  |  |  |
| SGLT2i | 22 | 214 367 | 0.10 | (0.07 to 0.16) | -0.12 | (-0.19 to -0.04) | 0.45 | (0.28 to 0.75) |
| DPP4i | 51 | 229 651 | 0.22 | (0.17 to 0.29) | 0.00 | (Ref) | 1.00 | (Ref) |
|  |  |  |  |  |  |  |  |  |
| **History of Asthma** |  |  |  |  |  |  |  |  |
| **Respiratory events^†^** |  |  |  |  |  |  |  |  |
| SGLT2i (n=10 686) | 137 | 11 993 | 11.42 | (9.66 to 13.51) | -5.41 | (-8.42 to -2.39) | 0.67 | (0.54 to 0.83) |
| DPP4i (n=9718) | 200 | 11 883 | 16.83 | (14.65 to 19.33) | 0.00 | (Ref) | 1.00 | (Ref) |
| **Acute pulmonary edema** |  |  |  |  |  |  |  |  |
| SGLT2i | 4 | 12 096 | 0.33 | (0.12 to 0.88) | -0.75 | (-1.41 to -0.08) | 0.30 | (0.10 to 0.93) |
| DPP4i | 13 | 12 081 | 1.08 | (0.62 to 1.85) | 0.00 | (Ref) | 1.00 | (Ref) |
| **ARDS** |  |  |  |  |  |  |  |  |
| SGLT2i | 1 | 12 101 | 0.08 | (0.01 to 0.59) | -0.00 | (-0.23 to 0.23) | 0.93 | (0.06 to 14.79) |
| DPP4i | 1 | 12 088 | 0.08 | (0.01 to 0.59) | 0.00 | (Ref) | 1.00 | (Ref) |
| **Pneumonia** |  |  |  |  |  |  |  |  |
| SGLT2i | 129 | 11 998 | 10.75 | (9.05 to 12.78) | -4.89 | (-7.80 to -1.97) | 0.67 | (0.54 to 0.84) |
| DPP4i | 186 | 11 894 | 15.64 | (13.55 to 18.06) | 0.00 | (Ref) | 1.00 | (Ref) |
| **Respiratory failure** |  |  |  |  |  |  |  |  |
| SGLT2i | 6 | 12 100 | 0.50 | (0.22 to 1.10) | -0.33 | (-0.98 to 0.32) | 0.62 | (0.22 to 1.71) |
| DPP4i | 10 | 12 080 | 0.83 | (0.45 to 1.54) | 0.00 | (Ref) | 1.00 | (Ref) |

^*^Users of SGLT2is were propensity-score matched to users of DPP4is in a 1:1 ratio.

^†^Defined as a composite end point of acute pulmonary edema, acute respiratory distress syndrome, pneumonia, or respiratory failure.

ARDS, acute respiratory pulmonary syndrome; DPP4i, dipeptidyl peptidase-4 inhibitor; SGLT2i, sodium glucose cotransporter 2 inhibitor

**Table S8** Stratified analysis on COPD history for the association between SGLT2is and risk of respiratory events.

|  | **No of events** | **Person-years** | **Incidence rate per 1000 person-years** | | **Rate difference per 1000 person-years (95% CI)** | | **Hazard ratio^*^ (95% CI)** | |
| --- | --- | --- | --- | --- | --- | --- | --- | --- |
|  |  |  |  |  |  |  |  |  |
| **No History of COPD** |  |  |  |  |  |  |  |  |
| **Respiratory events^†^** |  |  |  |  |  |  |  |  |
| SGLT2i (n=197 131) | 908 | 216 261 | 4.20 | (3.93 to 4.48) | -2.79 | (-3.22 to -2.35) | 0.60 | (0.55 to 0.65) |
| DPP4i (n=197 511) | 1608 | 230 243 | 6.98 | (6.65 to 7.33) | 0.00 | (Ref) | 1.00 | (Ref) |
| **Acute pulmonary edema** |  |  |  |  |  |  |  |  |
| SGLT2i | 21 | 217 059 | 0.10 | (0.06 to 0.15) | -0.21 | (-0.29 to -0.13) | 0.31 | (0.19 to 0.51) |
| DPP4i | 71 | 231 740 | 0.31 | (0.24 to 0.39) | 0.00 | (Ref) | 1.00 | (Ref) |
| **ARDS** |  |  |  |  |  |  |  |  |
| SGLT2i | 7 | 217 064 | 0.03 | (0.02 to 0.07) | -0.04 | (-0.08 to 0.00) | 0.46 | (0.19 to 1.13) |
| DPP4i | 16 | 231 797 | 0.07 | (0.04 to 0.11) | 0.00 | (Ref) | 1.00 | (Ref) |
| **Pneumonia** |  |  |  |  |  |  |  |  |
| SGLT2i | 873 | 216 278 | 4.04 | (3.78 to 4.31) | -2.55 | (-2.97 to -2.12) | 0.61 | (0.56 to 0.66) |
| DPP4i | 1516 | 230 308 | 6.58 | (6.26 to 6.92) | 0.00 | (Ref) | 1.00 | (Ref) |
| **Respiratory failure** |  |  |  |  |  |  |  |  |
| SGLT2i | 24 | 217 054 | 0.11 | (0.07 to 0.16) | -0.11 | (-0.19 to -0.04) | 0.49 | (0.30 to 0.79) |
| DPP4i | 52 | 231 773 | 0.22 | (0.17 to 0.29) | 0.00 | (Ref) | 1.00 | (Ref) |
|  |  |  |  |  |  |  |  |  |
| **History of COPD** |  |  |  |  |  |  |  |  |
| **Respiratory events^†^** |  |  |  |  |  |  |  |  |
| SGLT2i (n=8403) | 117 | 9322 | 12.55 | (10.47 to 15.04) | -8.12 | (-11.8 to -4.48) | 0.59 | (0.47 to 0.75) |
| DPP4i (n=8023) | 202 | 9770 | 20.67 | (18.01 to 23.73) | 0.00 | (Ref) | 1.00 | (Ref) |
| **Acute pulmonary edema** |  |  |  |  |  |  |  |  |
| SGLT2i | 5 | 9409 | 0.53 | (0.22 to 1.28) | -0.17 | (-0.87 to 0.53) | 0.74 | (0.23 to 2.33) |
| DPP4i | 7 | 9952 | 0.70 | (0.34 to 1.48) | 0.00 | (Ref) | 1.00 | (Ref) |
| **ARDS** |  |  |  |  |  |  |  |  |
| SGLT2i | 0 | 9414 | NA |  | -0.10 | (-0.30 to 0.10) |  | NA |
| DPP4i | 1 | 9960 | 0.10 | (0.01 to 0.71) | 0.00 | (Ref) | 1.00 | (Ref) |
| **Pneumonia** |  |  |  |  |  |  |  |  |
| SGLT2i | 111 | 9327 | 11.90 | (9.88 to 14.33) | -7.52 | (-11.1 to -3.98) | 0.60 | (0.47 to 0.76) |
| DPP4i | 190 | 9782 | 19.42 | (16.85 to 22.39) | 0.00 | (Ref) | 1.00 | (Ref) |
| **Respiratory failure** |  |  |  |  |  |  |  |  |
| SGLT2i | 4 | 9412 | 0.42 | (0.16 to 1.13) | -0.48 | (-1.20 to 0.24) | 0.47 | (0.14 to 1.52) |
| DPP4i | 9 | 9957 | 0.90 | (0.47 to 1.74) | 0.00 | (Ref) | 1.00 | (Ref) |

^*^Users of SGLT2is were propensity-score matched to users of DPP4is in a 1:1 ratio.

^†^Defined as a composite end point of acute pulmonary edema, acute respiratory distress syndrome, pneumonia, or respiratory failure.

ARDS, acute respiratory pulmonary syndrome; COPD, chronic obstructive pulmonary disease; DPP4i, dipeptidyl peptidase-4 inhibitor; SGLT2i, sodium glucose cotransporter 2 inhibitor

**Table S9** Stratified analysis on CVD history for the association between SGLT2is and risk of respiratory events.

|  | **No of events** | **Person-years** | **Incidence rate per 1000 person-years** | | **Rate difference per 1000 person-years (95% CI)** | | **Hazard ratio^*^ (95% CI)** | |
| --- | --- | --- | --- | --- | --- | --- | --- | --- |
|  |  |  |  |  |  |  |  |  |
| **No History of CVD** |  |  |  |  |  |  |  |  |
| **Respiratory events^†^** |  |  |  |  |  |  |  |  |
| SGLT2i (n=102 781) | 340 | 101 199 | 3.36 | (3.02 to 3.74) | -2.08 | (-2.65 to -1.51) | 0.61 | (0.54 to 0.70) |
| DPP4i (n=105 769) | 583 | 107 178 | 5.44 | (5.02 to 5.90) | 0.00 | (Ref) | 1.00 | (Ref) |
| **Acute pulmonary edema** |  |  |  |  |  |  |  |  |
| SGLT2i | 4 | 101 485 | 0.04 | (0.01 to 0.11) | -0.14 | (-0.23 to -0.05) | 0.22 | (0.08 to 0.66) |
| DPP4i | 19 | 107 735 | 0.18 | (0.11 to 0.28) | 0.00 | (Ref) | 1.00 | (Ref) |
| **ARDS** |  |  |  |  |  |  |  |  |
| SGLT2i | 2 | 101 485 | 0.02 | (0.00 to 0.08) | -0.05 | (-0.10 to 0.01) | 0.30 | (0.06 to 1.47) |
| DPP4i | 7 | 107 749 | 0.06 | (0.03 to 0.14) | 0.00 | (Ref) | 1.00 | (Ref) |
| **Pneumonia** |  |  |  |  |  |  |  |  |
| SGLT2i | 331 | 101 201 | 3.27 | (2.94 to 3.64) | -1.92 | (-2.47 to -1.36) | 0.62 | (0.55 to 0.72) |
| DPP4i | 556 | 107 199 | 5.19 | (4.77 to 5.64) | 0.00 | (Ref) | 1.00 | (Ref) |
| **Respiratory failure** |  |  |  |  |  |  |  |  |
| SGLT2i | 9 | 101 482 | 0.09 | (0.05 to 0.17) | -0.04 | (-0.13 to 0.05) | 0.68 | (0.29 to 1.56) |
| DPP4i | 14 | 107 742 | 0.13 | (0.08 to 0.22) | 0.00 | (Ref) | 1.00 | (Ref) |
|  |  |  |  |  |  |  |  |  |
| **History of CVD** |  |  |  |  |  |  |  |  |
| **Respiratory events^†^** |  |  |  |  |  |  |  |  |
| SGLT2i (n=102 753) | 685 | 124 384 | 5.51 | (5.11 to 5.94) | -3.73 | (-4.39 to -3.07) | 0.59 | (0.54 to 0.65) |
| DPP4i (n=99 765) | 1227 | 132 835 | 9.24 | (8.73 to 9.77) | 0.00 | (Ref) | 1.00 | (Ref) |
| **Acute pulmonary edema** |  |  |  |  |  |  |  |  |
| SGLT2i | 22 | 124 983 | 0.18 | (0.12 to 0.27) | -0.26 | (-0.40 to -0.13) | 0.40 | (0.24 to 0.65) |
| DPP4i | 59 | 133 957 | 0.44 | (0.34 to 0.57) | 0.00 | (Ref) | 1.00 | (Ref) |
| **ARDS** |  |  |  |  |  |  |  |  |
| SGLT2i | 5 | 124 993 | 0.04 | (0.02 to 0.10) | -0.03 | (-0.09 to 0.02) | 0.52 | (0.18 to 1.52) |
| DPP4i | 10 | 134 009 | 0.07 | (0.04 to 0.14) | 0.00 | (Ref) | 1.00 | (Ref) |
| **Pneumonia** |  |  |  |  |  |  |  |  |
| SGLT2i | 653 | 124 404 | 5.25 | (4.86 to 5.67) | -3.40 | (-4.05 to -2.76) | 0.60 | (0.54 to 0.66) |
| DPP4i | 1150 | 132 892 | 8.65 | (8.17 to 9.17) | 0.00 | (Ref) | 1.00 | (Ref) |
| **Respiratory failure** |  |  |  |  |  |  |  |  |
| SGLT2i | 19 | 124 985 | 0.15 | (0.10 to 0.24) | -0.20 | (-0.32 to -0.08) | 0.43 | (0.25 to 0.73) |
| DPP4i | 47 | 133 989 | 0.35 | (0.26 to 0.47) | 0.00 | (Ref) | 1.00 | (Ref) |

^*^Users of SGLT2is were propensity-score matched to users of DPP4is in a 1:1 ratio.

^†^Defined as a composite end point of acute pulmonary edema, acute respiratory distress syndrome, pneumonia, or respiratory failure.

ARDS, acute respiratory pulmonary syndrome; CVD, cardiovascular disease; DPP4i, dipeptidyl peptidase-4 inhibitor; SGLT2i, sodium glucose cotransporter 2 inhibitor

**Table S10** Stratified analysis on heart failure history for the association between SGLT2is and risk of respiratory events.

|  | **No of events** | **Person-years** | **Incidence rate per 1000 person-years** | | **Rate difference per 1000 person-years (95% CI)** | | **Hazard ratio^*^ (95% CI)** | |
| --- | --- | --- | --- | --- | --- | --- | --- | --- |
|  |  |  |  |  |  |  |  |  |
| **No History of Heart Failure** |  |  |  |  |  |  |  |  |
| **Respiratory events^†^** |  |  |  |  |  |  |  |  |
| SGLT2i (n=202 119) | 970 | 222 034 | 4.37 | (4.10 to 4.65) | -2.93 | (-3.37 to -2.49) | 0.59 | (0.55 to 0.64) |
| DPP4i (n=202 827) | 1729 | 236 969 | 7.30 | (6.96 to 7.65) | 0.00 | (Ref) | 1.00 | (Ref) |
| **Acute pulmonary edema** |  |  |  |  |  |  |  |  |
| SGLT2i | 24 | 222 878 | 0.11 | (0.07 to 0.16) | -0.17 | (-0.25 to -0.09) | 0.39 | (0.24 to 0.62) |
| DPP4i | 66 | 238 606 | 0.28 | (0.22 to 0.35) | 0.00 | (Ref) | 1.00 | (Ref) |
| **ARDS** |  |  |  |  |  |  |  |  |
| SGLT2i | 7 | 222 888 | 0.03 | (0.01 to 0.07) | -0.03 | (-0.07 to 0.01) | 0.50 | (0.20 to 1.22) |
| DPP4i | 15 | 238 664 | 0.06 | (0.04 to 0.10) | 0.00 | (Ref) | 1.00 | (Ref) |
| **Pneumonia** |  |  |  |  |  |  |  |  |
| SGLT2i | 933 | 222 055 | 4.20 | (3.94 to 4.48) | -2.71 | (-3.14 to -2.28) | 0.60 | (0.55 to 0.65) |
| DPP4i | 1639 | 237 035 | 6.91 | (6.59 to 7.26) | 0.00 | (Ref) | 1.00 | (Ref) |
| **Respiratory failure** |  |  |  |  |  |  |  |  |
| SGLT2i | 26 | 222 878 | 0.12 | (0.08 to 0.17) | -0.12 | (-0.20 to -0.05) | 0.48 | (0.30 to 0.77) |
| DPP4i | 57 | 238 636 | 0.24 | (0.18 to 0.31) | 0.00 | (Ref) | 1.00 | (Ref) |
|  |  |  |  |  |  |  |  |  |
| **History of Heart Failure** |  |  |  |  |  |  |  |  |
| **Respiratory events^†^** |  |  |  |  |  |  |  |  |
| SGLT2i (n=3415) | 55 | 3549 | 15.50 | (11.90 to 20.12) | -11.11 | (-18.20 to -4.01) | 0.58 | (0.41 to 0.81) |
| DPP4i (n=2707) | 81 | 3044 | 26.61 | (21.40 to 33.87) | 0.00 | (Ref) | 1.00 | (Ref) |
| **Acute pulmonary edema** |  |  |  |  |  |  |  |  |
| SGLT2i | 2 | 3590 | 0.56 | (0.14 to 2.23) | -3.33 | (-5.66 to -1.00) | 0.14 | (0.03 to 0.63) |
| DPP4i | 12 | 3086 | 3.89 | (2.21 to 6.85) | 0.00 | (Ref) | 1.00 | (Ref) |
| **ARDS** |  |  |  |  |  |  |  |  |
| SGLT2i | 0 | 3590 | 0.00 | NA | NA |  | NA |  |
| DPP4i | 2 | 3094 | 0.65 | (0.16 to 2.58) | 0.00 | (Ref) | 1.00 | (Ref) |
| **Pneumonia** |  |  |  |  |  |  |  |  |
| SGLT2i | 51 | 3549 | 14.37 | (10.92 to 18.55) | -7.56 | (-14.1 to -0.99) | 0.65 | (0.45 to 0.93) |
| DPP4i | 67 | 3056 | 21.93 | (17.26 to 27.38) | 0.00 | (Ref) | 1.00 | (Ref) |
| **Respiratory failure** |  |  |  |  |  |  |  |  |
| SGLT2i | 2 | 3589 | 0.56 | (0.14 to 2.33) | -0.74 | (-2.22 to 0.75) | 0.43 | (0.08 to 2.37) |
| DPP4i | 4 | 3095 | 1.29 | (0.49 to 3.44) | 0.00 | (Ref) | 1.00 | (Ref) |

^*^Users of SGLT2is were propensity-score matched to users of DPP4is in a 1:1 ratio.

^†^Defined as a composite end point of acute pulmonary edema, acute respiratory distress syndrome, pneumonia, or respiratory failure.

ARDS, acute respiratory pulmonary syndrome; CKD, chronic kidney disease; DPP4i, dipeptidyl peptidase-4 inhibitor; SGLT2i, sodium glucose cotransporter 2 inhibitor

**Table S11** Stratified analysis on CKD history for the association between SGLT2is and risk of respiratory events.

|  | **No of events** | **Person-years** | **Incidence rate per 1000 person-years** | | **Rate difference per 1000 person-years (95% CI)** | | **Hazard ratio^*^ (95% CI)** | |
| --- | --- | --- | --- | --- | --- | --- | --- | --- |
|  |  |  |  |  |  |  |  |  |
| **No History of CKD** |  |  |  |  |  |  |  |  |
| **Respiratory events^†^** |  |  |  |  |  |  |  |  |
| SGLT2i (n=197 261) | 954 | 215 647 | 4.42 | (4.15 to 4.71) | -2.85 | (-3.29 to -2.40) | 0.60 | (0.56 to 0.65) |
| DPP4i (n=198 124) | 1673 | 230 083 | 7.27 | (6.93 to 7.63) | 0.00 | (Ref) | 1.00 | (Ref) |
| **Acute pulmonary edema** |  |  |  |  |  |  |  |  |
| SGLT2i | 23 | 216 455 | 0.11 | (0.07 to 0.16) | -0.17 | (-0.26 to -0.09) | 0.38 | (0.23 to 0.61) |
| DPP4i | 65 | 231 661 | 0.28 | (0.22 to 0.36) | 0.00 | (Ref) | 1.00 | (Ref) |
| **ARDS** |  |  |  |  |  |  |  |  |
| SGLT2i | 5 | 216 461 | 0.02 | (0.01 to 0.06) | -0.05 | (-0.09 to -0.01) | 0.31 | (0.11 to 0.84) |
| DPP4i | 17 | 231 714 | 0.07 | (0.05 to 0.12) | 0.00 | (Ref) | 1.00 | (Ref) |
| **Pneumonia** |  |  |  |  |  |  |  |  |
| SGLT2i | 918 | 215 663 | 4.26 | (3.99 to 4.54) | -2.61 | (-3.04 to -2.17) | 0.61 | (0.57 to 0.67) |
| DPP4i | 1580 | 230 150 | 6.87 | (6.53 to 7.21) | 0.00 | (Ref) | 1.00 | (Ref) |
| **Respiratory failure** |  |  |  |  |  |  |  |  |
| SGLT2i | 27 | 216 451 | 0.12 | (0.09 to 0.18) | -0.11 | (-0.19 to -0.03) | 0.53 | (0.33 to 0.84) |
| DPP4i | 54 | 231 688 | 0.23 | (0.18 to 0.30) | 0.00 | (Ref) | 1.00 | (Ref) |
|  |  |  |  |  |  |  |  |  |
| **History of CKD** |  |  |  |  |  |  |  |  |
| **Respiratory events^†^** |  |  |  |  |  |  |  |  |
| SGLT2i (n=8273) | 71 | 9936 | 7.15 | (5.66 to 9.02) | -6.65 | (-9.50 to -3.80) | 0.51 | (0.38 to 0.68) |
| DPP4i (n=7410) | 137 | 9930 | 13.80 | (11.67 to 16.56) | 0.00 | (Ref) | 1.00 | (Ref) |
| **Acute pulmonary edema** |  |  |  |  |  |  |  |  |
| SGLT2i | 3 | 10 012 | 0.30 | (0.10 to 0.93) | -1.00 | (-1.78 to -0.21) | 0.23 | (0.07 to 0.81) |
| DPP4i | 13 | 10 031 | 1.30 | (0.75 to 2.23) | 0.00 | (Ref) | 1.00 | (Ref) |
| **ARDS** |  |  |  |  |  |  |  |  |
| SGLT2i | 2 | 10 016 | 0.20 | (0.05 to 0.80) |  | NA |  | NA |
| DPP4i | 0 | 10 044 |  | NA | 0.00 | (Ref) | 1.00 | (Ref) |
| **Pneumonia** |  |  |  |  |  |  |  |  |
| SGLT2i | 66 | 9941 | 6.64 | (5.22 to 8.45) | -6.04 | (-8.77 to -3.30) | 0.52 | (0.38 to 0.69) |
| DPP4i | 126 | 9940 | 12.68 | (10.65 to 15.35) | 0.00 | (Ref) | 1.00 | (Ref) |
| **Respiratory failure** |  |  |  |  |  |  |  |  |
| SGLT2i | 1 | 10 017 | 0.10 | (0.01 to 0.71) | -0.60 | (-1.15 to -0.05) | 0.15 | (0.02 to 1.18) |
| DPP4i | 7 | 10 042 | 0.70 | (0.33 to 1.46) | 0.00 | (Ref) | 1.00 | (Ref) |

^*^Users of SGLT2is were propensity-score matched to users of DPP4is in a 1:1 ratio.

^†^Defined as a composite end point of acute pulmonary edema, acute respiratory distress syndrome, pneumonia, or respiratory failure.

ARDS, acute respiratory pulmonary syndrome; CKD, chronic kidney disease; DPP4i, dipeptidyl peptidase-4 inhibitor; SGLT2i, sodium glucose cotransporter 2 inhibitor

**Table S12** Association between SGLT2is and risk of respiratory events, stratified on follow-up duration.

|  | **No of events** | **Person-years** | **Incidence rate per 1000 person-years** | | **Rate difference per 1000 person-years (95% CI)** | | **Hazard ratio^*^ (95% CI)** | |
| --- | --- | --- | --- | --- | --- | --- | --- | --- |
|  |  |  |  |  |  |  |  |  |
| **Duration of follow-up <1 year** |  |  |  |  |  |  |  |  |
| **Respiratory events^†^** |  |  |  |  |  |  |  |  |
| SGLT2i (n=126 819) | 660 | 47 079 | 14.02 | (12.99 to 15.37) | -9.24 | (-11.0 to -7.49) | 0.60 | (0.55 to 0.66) |
| DPP4i (n=124 111) | 1088 | 46 784 | 23.26 | (21.91 to 24.76) | 0.00 | (Ref) | 1.00 | (Ref) |
| **Acute pulmonary edema** |  |  |  |  |  |  |  |  |
| SGLT2i | 20 | 47 604 | 0.42 | (0.27 to 0.65) | -0.42 | (-0.73 to -0.10) | 0.50 | (0.29 to 0.86) |
| DPP4i | 40 | 47 796 | 0.84 | (0.61 to 1.14) | 0.00 | (Ref) | 1.00 | (Ref) |
| **ARDS** |  |  |  |  |  |  |  |  |
| SGLT2i | 5 | 47 612 | 0.11 | (0.04 to 0.25) | -0.10 | (-0.26 to 0.05) | 0.50 | (0.17 to 1.46) |
| DPP4i | 10 | 47 834 | 0.21 | (0.11 to 0.39) | 0.00 | (Ref) | 1.00 | (Ref) |
| **Pneumonia** |  |  |  |  |  |  |  |  |
| SGLT2i | 633 | 47 092 | 13.44 | (12.43 to 14.88) | -8.51 | (-10.2 to -6.81) | 0.61 | (0.55 to 0.68) |
| DPP4i | 1028 | 46 829 | 21.95 | (20.65 to 23.14) | 0.00 | (Ref) | 1.00 | (Ref) |
| **Respiratory failure** |  |  |  |  |  |  |  |  |
| SGLT2i | 15 | 47 605 | 0.32 | (0.19 to 0.52) | -0.48 | (-0.78 to -0.18) | 0.40 | (0.22 to 0.72) |
| DPP4i | 38 | 47 820 | 0.79 | (0.58 to 1.09) | 0.00 | (Ref) | 1.00 | (Ref) |
|  |  |  |  |  |  |  |  |  |
| **Duration of follow-up ≥1 year** |  |  |  |  |  |  |  |  |
| **Respiratory events^†^** |  |  |  |  |  |  |  |  |
| SGLT2i (n=78 715) | 365 | 178 507 | 2.04 | (1.85 to 2.27) | -1.69 | (-2.04 to -1.35) | 0.56 | (0.50 to 0.64) |
| DPP4i (n=81 423) | 722 | 193 229 | 3.74 | (3.47 to 4.02) | 0.00 | (Ref) | 1.00 | (Ref) |
| **Acute pulmonary edema** |  |  |  |  |  |  |  |  |
| SGLT2i | 6 | 178 864 | 0.03 | (0.02 to 0.07) | -0.16 | (-0.23 to -0.09) | 0.18 | (0.08 to 0.42) |
| DPP4i | 38 | 193 896 | 0.20 | (0.14 to 0.27) | 0.00 | (Ref) | 1.00 | (Ref) |
| **ARDS** |  |  |  |  |  |  |  |  |
| SGLT2i | 2 | 178 866 | 0.01 | (0.00 to 0.04) | -0.02 | (-0.06 to 0.01) | 0.32 | (0.07 to 1.54) |
| DPP4i | 7 | 193 924 | 0.04 | (0.02 to 0.08) | 0.00 | (Ref) | 1.00 | (Ref) |
| **Pneumonia** |  |  |  |  |  |  |  |  |
| SGLT2i | 351 | 178 513 | 1.97 | (1.77 to 2.18) | -1.54 | (-1.88 to -1.21) | 0.58 | (0.51 to 0.66) |
| DPP4i | 678 | 193 261 | 3.51 | (3.25 to 3.78) | 0.00 | (Ref) | 1.00 | (Ref) |
| **Respiratory failure** |  |  |  |  |  |  |  |  |
| SGLT2i | 13 | 178 862 | 0.07 | (0.04 to 0.13) | -0.05 | (-0.11 to 0.02) | 0.63 | (0.32 to 1.24) |
| DPP4i | 23 | 193 911 | 0.12 | (0.08 to 0.18) | 0.00 | (Ref) | 1.00 | (Ref) |

^*^Users of SGLT2is were propensity-score matched to users of DPP4is in a 1:1 ratio.

^†^Defined as a composite end point of acute pulmonary edema, acute respiratory distress syndrome, pneumonia, or respiratory failure.

ARDS, acute respiratory pulmonary syndrome; CKD, chronic kidney disease; DPP4i, dipeptidyl peptidase-4 inhibitor; SGLT2i, sodium glucose cotransporter 2 inhibitor

**Table S13** Sensitivity analysis using an intention-to-treat definition.

|  | **No of events** | **Person-years** | **Incidence rate per 1000 person-years** | | **Rate difference per 1000 person-years (95% CI)** | | **Hazard ratio^*^ (95% CI)** | |
| --- | --- | --- | --- | --- | --- | --- | --- | --- |
|  |  |  |  |  |  |  |  |  |
| **Respiratory events^†^** |  |  |  |  |  |  |  |  |
| SGLT2i | 1132 | 179 875 | 6.29 | (5.94 to 6.67) | -2.65 | (-3.22 to -2.08) | 0.70 | (0.65 to 0.76) |
| DPP4i | 1611 | 180 094 | 8.95 | (8.52 to 9.39) | 0.00 | (Ref) | 1.00 | (Ref) |
| **Acute pulmonary edema** |  |  |  |  |  |  |  |  |
| SGLT2i | 34 | 180 406 | 0.19 | (0.13 to 0.26) | -0.10 | (-0.20 to 0.00) | 0.66 | (0.43 to 1.01) |
| DPP4i | 52 | 180 811 | 0.29 | (0.22 to 0.38) | 0.00 | (Ref) | 1.00 | (Ref) |
| **ARDS** |  |  |  |  |  |  |  |  |
| SGLT2i | 9 | 180 419 | 0.05 | (0.03 to 0.10) | -0.03 | (-0.08 to 0.02) | 0.64 | (0.28 to 1.49) |
| DPP4i | 14 | 180 832 | 0.08 | (0.05 to 0.13) | 0.00 | (Ref) | 1.00 | (Ref) |
| **Pneumonia** |  |  |  |  |  |  |  |  |
| SGLT2i | 1083 | 179 893 | 6.02 | (5.67 to 6.39) | -2.46 | (-3.02 to -1.91) | 0.71 | (0.66 to 0.77) |
| DPP4i | 1528 | 180 128 | 8.48 | (8.07 to 8.92) | 0.00 | (Ref) | 1.00 | (Ref) |
| **Respiratory failure** |  |  |  |  |  |  |  |  |
| SGLT2i | 32 | 180 413 | 0.18 | (0.13 to 0.25) | -0.15 | (-0.25 to -0.05) | 0.54 | (0.35 to 0.84) |
| DPP4i | 59 | 180 818 | 0.33 | (0.25 to 0.42) | 0.00 | (Ref) | 1.00 | (Ref) |

^*^Users of SGLT2is were propensity-score matched to users of DPP4is in a 1:1 ratio.

^†^Defined as a composite end point of acute pulmonary edema, acute respiratory distress syndrome, pneumonia, or respiratory failure.

ARDS, acute respiratory pulmonary syndrome; DPP4i, dipeptidyl peptidase-4 inhibitor; SGLT2i, sodium glucose cotransporter 2 inhibitor

**Table S14** Sensitivity analysis that treated in-hospital death as a competing event^‡^.

|  | **No of events** | **Person-years** | **Incidence rate per 1000 person-years** | | **Rate difference per 1000 person-years (95% CI)** | | **Hazard ratio^*^ (95% CI)** | |
| --- | --- | --- | --- | --- | --- | --- | --- | --- |
| **Respiratory events^†^** |  |  |  |  |  |  |  |  |
| SGLT2i | 1025 | 225 583 | 4.54 | (4.27 to 4.83) | -3.00 | (-3.44 to -2.55) | 0.60 | (0.55 to 0.64) |
| DPP4i | 1810 | 240 013 | 7.54 | (7.20 to 7.90) | 0.00 | (Ref) | 1.00 | (Ref) |
| **Acute pulmonary edema** |  |  |  |  |  |  |  |  |
| SGLT2i | 26 | 226 468 | 0.11 | (0.08 to 0.17) | -0.21 | (-0.29 to -0.12) | 0.36 | (0.23 to 0.55) |
| DPP4i | 78 | 241 692 | 0.32 | (0.26 to 0.40) | 0.00 | (Ref) | 1.00 | (Ref) |
| **ARDS** |  |  |  |  |  |  |  |  |
| SGLT2i | 7 | 226 478 | 0.03 | (0.01 to 0.06) | -0.04 | (-0.08 to 0.00) | 0.44 | (0.18 to 1.05) |
| DPP4i | 17 | 241 758 | 0.07 | (0.04 to 0.11) | 0.00 | (Ref) | 1.00 | (Ref) |
| **Pneumonia** |  |  |  |  |  |  |  |  |
| SGLT2i | 984 | 225 605 | 4.36 | (4.10 to 4.64) | -2.74 | (-3.18 to -2.31) | 0.61 | (0.56 to 0.66) |
| DPP4i | 1706 | 240 090 | 7.11 | (6.78 to 7.45) | 0.00 | (Ref) | 1.00 | (Ref) |
| **Respiratory failure** |  |  |  |  |  |  |  |  |
| SGLT2i | 28 | 226 467 | 0.12 | (0.09 to 0.18) | -0.13 | (-0.21 to -0.05) | 0.49 | (0.31 to 0.76) |
| DPP4i | 61 | 241 730 | 0.25 | (0.20 to 0.32) | 0.00 | (Ref) | 1.00 | (Ref) |

^*^Users of SGLT2is were propensity-score matched to users of DPP4is in a 1:1 ratio.

^†^Defined as a composite end point of acute pulmonary edema, acute respiratory distress syndrome, pneumonia, or respiratory failure.

^‡^In-hospital death was treated as a competing event and not a censoring event in the Fine-Gray subdistribution hazards models.

ARDS, acute respiratory distress syndrome; DPP4i, dipeptidyl peptidase-4 inhibitor; SGLT2i, sodium glucose cotransporter 2 inhibitor

**Table S15** Association between SGLT2is versus DPP4is and risk of negative and positive control outcomes.

|  | **No of events** | **Person-years** | **Incidence rate per 1000 person-years** | | **Rate difference per 1000 person-years (95% CI)** | | **Hazard ratio^*^ (95% CI)** | |
| --- | --- | --- | --- | --- | --- | --- | --- | --- |
| **Negative control outcome^†^** |  |  |  |  |  |  |  |  |
| **Herpes zoster virus reactivation** |  |  |  |  |  |  |  |  |
| SGLT2i | 5461 | 220 211 | 24.80 | (24.15 to 25.47) | 0.66 | (-0.25 to 1.57) | 1.02 | (0.98 to 1.06) |
| DPP4i | 5663 | 234 595 | 24.14 | (23.52 to 24.78) | 0.00 | (Ref) | 1.00 | (Ref) |
| **Positive control outcome**^‡^ |  |  |  |  |  |  |  |  |
| Hospitalization for heart failure |  |  |  |  |  |  |  |  |
| SGLT2i | 403 | 226 244 | 1.78 | (1.62 to 1.96) | -0.77 | (-1.03 to -0.50) | 0.69 | (0.61 to 0.78) |
| DPP4i | 615 | 241 296 | 2.55 | (2.36 to 2.76) | 0.00 | (Ref) | 1.00 | (Ref) |

^*^Users of SGLT2is were propensity-score matched to users of DPP4is in a 1:1 ratio.

^†^Expect a null association with SGLT2is versus DPP4is.

^‡^Expect a protective association with SGLT2is versus DPP4is.

DPP4i, dipeptidyl peptidase-4 inhibitor; SGLT2i, sodium glucose cotransporter 2 inhibitor

**Table S16** Sensitivity analysis that excluded patients with a history of insulin use alone.

|  | **No of events** | **Person-years** | **Incidence rate per 1000 person-years** | | **Rate difference per 1000 person-years (95% CI)** | | **Hazard ratio^*^ (95% CI)** | |
| --- | --- | --- | --- | --- | --- | --- | --- | --- |
| **Respiratory events^†^** |  |  |  |  |  |  |  |  |
| SGLT2i (n=198 404) | 957 | 218 153 | 4.39 | (4.12 to 4.67) | -3.35 | (-3.81 to -2.90) | 0.56 | (0.52 to 0.61) |
| DPP4i (n=198 404) | 1791 | 231 343 | 7.74 | (7.39 to 8.11) | 0.00 | (Ref) | 1.00 | (Ref) |
| **Acute pulmonary edema** |  |  |  |  |  |  |  |  |
| SGLT2i | 24 | 218 980 | 0.11 | (0.07 to 0.16) | -0.16 | (-0.24 to -0.08) | 0.41 | (0.25 to 0.65) |
| DPP4i | 63 | 232 994 | 0.27 | (0.21 to 0.35) | 0.00 | (Ref) | 1.00 | (Ref) |
| **ARDS** |  |  |  |  |  |  |  |  |
| SGLT2i | 7 | 218 990 | 0.03 | (0.02 to 0.07) | -0.04 | (-0.08 to 0.00) | 0.48 | (0.20 to 1.16) |
| DPP4i | 16 | 233 019 | 0.07 | (0.04 to 0.11) | 0.00 | (Ref) | 1.00 | (Ref) |
| **Pneumonia** |  |  |  |  |  |  |  |  |
| SGLT2i | 917 | 218 175 | 4.20 | (3.94 to 4.48) | -3.11 | (-3.55 to -2.67) | 0.57 | (0.53 to 0.62) |
| DPP4i | 1692 | 231 390 | 7.31 | (6.97 to 7.67) | 0.00 | (Ref) | 1.00 | (Ref) |
| **Respiratory failure** |  |  |  |  |  |  |  |  |
| SGLT2i | 28 | 218 979 | 0.13 | (0.09 to 0.19) | -0.22 | (-0.31 to -0.13) | 0.37 | (0.24 to 0.57) |
| DPP4i | 81 | 233 009 | 0.35 | (0.28 to 0.43) | 0.00 | (Ref) | 1.00 | (Ref) |

^*^Users of SGLT2is were propensity-score matched to users of DPP4is in a 1:1 ratio.

^†^Defined as a composite end point of acute pulmonary edema, acute respiratory distress syndrome, pneumonia, or respiratory failure.

ARDS, acute respiratory distress syndrome; DPP4i, dipeptidyl peptidase-4 inhibitor; SGLT2i, sodium glucose cotransporter 2 inhibitor

**Table S17** Sensitivity analysis that varied the grace period duration.

|  | **No of events** | **Person-years** | **Incidence rate per 1000 person-years** | | **Rate difference per 1000 person-years (95% CI)** | | **Hazard ratio^*^ (95% CI)** | |
| --- | --- | --- | --- | --- | --- | --- | --- | --- |
|  |  |  |  |  |  |  |  |  |
| **Grace period of 0 days** |  |  |  |  |  |  |  |  |
| **Respiratory events^†^** |  |  |  |  |  |  |  |  |
| SGLT2i (n=205 534) | 204 | 33 184 | 6.15 | (5.66 to 7.05) | -2.20 | (3.56 to -0.84) | 0.77 | (0.63 to 0.92) |
| DPP4i (n=205 534) | 233 | 27 911 | 8.35 | (7.34 to 9.39) | 0.00 | (Ref) | 1.00 | (Ref) |
| **Acute pulmonary edema** |  |  |  |  |  |  |  |  |
| SGLT2i | 7 | 33 207 | 0.21 | (0.10 to 0.44) | -0.00 | (-0.24 to 0.23) | 1.05 | (0.35 to 3.14) |
| DPP4i | 6 | 27 933 | 0.21 | (0.10 to 0.48) | 0.00 | (Ref) | 1.00 | (Ref) |
| **ARDS** |  |  |  |  |  |  |  |  |
| SGLT2i | 3 | 33 207 | 0.09 | (0.03 to 0.28) | -0.09 | (-0.28 to 0.10) | 0.51 | (0.12 to 2.15) |
| DPP4i | 5 | 27 934 | 0.18 | (0.07 to 0.43) | 0.00 | (Ref) | 1.00 | (Ref) |
| **Pneumonia** |  |  |  |  |  |  |  |  |
| SGLT2i | 195 | 33 184 | 5.88 | (5.11 to 6.76) | -2.08 | (-3.41 to -0.75) | 0.77 | (0.63 to 0.93) |
| DPP4i | 222 | 27 912 | 7.95 | (6.97 to 9.07) | 0.00 | (Ref) | 1.00 | (Ref) |
| **Respiratory failure** |  |  |  |  |  |  |  |  |
| SGLT2i | 2 | 33 207 | 0.06 | (0.02 to 0.24) | -0.12 | (-0.30 to 0.06) | 0.35 | (0.07 to 1.80) |
| DPP4i | 5 | 27 934 | 0.18 | (0.07 to 0.43) | 0.00 | (Ref) | 1.00 | (Ref) |
|  |  |  |  |  |  |  |  |  |
| **Grace period of 60 days** |  |  |  |  |  |  |  |  |
| **Respiratory events^†^** |  |  |  |  |  |  |  |  |
| SGLT2i (n=205 534) | 1193 | 255 420 | 4.67 | (4.41 to 4.94) | -3.23 | (-3.65 to -2.81) | 0.58 | (0.54 to 0.63) |
| DPP4i (n=205 534) | 2212 | 279 949 | 7.90 | (7.58 to 8.24) | 0.00 | (Ref) | 1.00 | (Ref) |
| **Acute pulmonary edema** |  |  |  |  |  |  |  |  |
| SGLT2i | 27 | 256 543 | 0.11 | (0.07 to 0.15) | -0.25 | (-0.33 to -0.17) | 0.30 | (0.19 to 0.45) |
| DPP4i | 100 | 282 228 | 0.35 | (0.29 to 0.43) | 0.00 | (Ref) | 1.00 | (Ref) |
| **ARDS** |  |  |  |  |  |  |  |  |
| SGLT2i | 10 | 256 555 | 0.04 | (0.02 to 0.07) | -0.04 | (-0.08 to 0.00) | 0.52 | (0.24 to 1.10) |
| DPP4i | 21 | 282 314 | 0.07 | (0.05 to 0.11) | 0.00 | (Ref) | 1.00 | (Ref) |
| **Pneumonia** |  |  |  |  |  |  |  |  |
| SGLT2i | 1146 | 255 448 | 4.49 | (4.23 to 4.75) | -2.96 | (-3.37 to -2.54) | 0.60 | (0.55 to 0.64) |
| DPP4i | 2084 | 280 056 | 7.44 | (7.13 to 7.77) | 0.00 | (Ref) | 1.00 | (Ref) |
| **Respiratory failure** |  |  |  |  |  |  |  |  |
| SGLT2i | 34 | 256 540 | 0.13 | (0.09 to 0.19) | -0.15 | (-0.22 to -0.07) | 0.47 | (0.31 to 0.70) |
| DPP4i | 79 | 282 270 | 0.28 | (0.22 to 0.35) | 0.00 | (Ref) | 1.00 | (Ref) |

^*^Users of SGLT2is were propensity-score matched to users of DPP4is in a 1:1 ratio.

^†^Defined as a composite end point of acute pulmonary edema, acute respiratory distress syndrome, pneumonia, or respiratory failure.

ARDS, acute respiratory pulmonary syndrome; CKD, chronic kidney disease; DPP4i, dipeptidyl peptidase-4 inhibitor; SGLT2i, sodium glucose cotransporter 2 inhibitor

**Table S18** Sensitivity analysis that used diagnosis codes recorded in the primary position.

|  | **No of events** | **Person-years** | **Incidence rate per 1000 person-years** | | **Rate difference per 1000 person-years (95% CI)** | | **Hazard ratio^*^ (95% CI)** | |
| --- | --- | --- | --- | --- | --- | --- | --- | --- |
|  |  |  |  |  |  |  |  |  |
| **Respiratory events^†^** |  |  |  |  |  |  |  |  |
| SGLT2i (n=205 534) | 815 | 225 709 | 3.61 | (3.37 to 3.87) | -2.12 | (-2.51 to -1.73) | 0.62 | (0.57 to 0.68) |
| DPP4i (n=205 534) | 1377 | 240 358 | 5.73 | (5.43 to 6.04) | 0.00 | (Ref) | 1.00 | (Ref) |
| **Acute pulmonary edema** |  |  |  |  |  |  |  |  |
| SGLT2i | 13 | 226 472 | 0.06 | (0.03 to 0.10) | -0.11 | (-0.17 to -0.05) | 0.35 | (0.19 to 0.65) |
| DPP4i | 40 | 241 723 | 0.17 | (0.12 to 0.23) | 0.00 | (Ref) | 1.00 | (Ref) |
| **ARDS** |  |  |  |  |  |  |  |  |
| SGLT2i | 5 | 226 478 | 0.02 | (0.01 to 0.05) | -0.01 | (-0.04 to 0.02) | 0.66 | (0.22 to 2.02) |
| DPP4i | 8 | 241 759 | 0.03 | (0.02 to 0.07) | 0.00 | (Ref) | 1.00 | (Ref) |
| **Pneumonia** |  |  |  |  |  |  |  |  |
| SGLT2i | 782 | 225 720 | 3.46 | (3.23 to 3.72) | -2.00 | (-2.38 to -1.62) | 0.63 | (0.57 to 0.69) |
| DPP4i | 1314 | 240 407 | 5.47 | (5.18 to 5.77) | 0.00 | (Ref) | 1.00 | (Ref) |
| **Respiratory failure** |  |  |  |  |  |  |  |  |
| SGLT2i | 19 | 226 474 | 0.08 | (0.05 to 0.13) | -0.04 | (-0.09 to 0.02) | 0.70 | (0.39 to 1.25) |
| DPP4i | 29 | 241 745 | 0.12 | (0.08 to 0.17) | 0.00 | (Ref) | 1.00 | (Ref) |

^*^Users of SGLT2is were propensity-score matched to users of DPP4is in a 1:1 ratio.

^†^Defined as a composite end point of acute pulmonary edema, acute respiratory distress syndrome, pneumonia, or respiratory failure.

ARDS, acute respiratory pulmonary syndrome; CKD, chronic kidney disease; DPP4i, dipeptidyl peptidase-4 inhibitor; SGLT2i, sodium glucose cotransporter 2 inhibitor

**Table S19** Sensitivity analysis that extended the baseline period.

|  | **No of events** | **Person-years** | **Incidence rate per 1000 person-years** | | **Rate difference per 1000 person-years (95% CI)** | | **Hazard ratio^*^ (95% CI)** | |
| --- | --- | --- | --- | --- | --- | --- | --- | --- |
|  |  |  |  |  |  |  |  |  |
| **Respiratory events^†^** |  |  |  |  |  |  |  |  |
| SGLT2i (n=172 615) | 740 | 170 039 | 4.35 | (4.05 to 4.68) | -3.28 | (-3.80 to -2.77) | 0.57 | (0.52 to 0.62) |
| DPP4i (n=172 615) | 1340 | 175 505 | 7.64 | (7.24 to 8.06) | 0.00 | (Ref) | 1.00 | (Ref) |
| **Acute pulmonary edema** |  |  |  |  |  |  |  |  |
| SGLT2i | 19 | 170 562 | 0.11 | (0.07 to 0.17) | -0.19 | (-0.29 to -0.10) | 0.36 | (0.22 to 0.61) |
| DPP4i | 54 | 176 514 | 0.31 | (0.23 to 0.40) | 0.00 | (Ref) | 1.00 | (Ref) |
| **ARDS** |  |  |  |  |  |  |  |  |
| SGLT2i | 7 | 170 565 | 0.04 | (0.02 to 0.09) | -0.03 | (-0.08 to 0.02) | 0.60 | (0.24 to 1.53) |
| DPP4i | 12 | 176 541 | 0.07 | (0.04 to 0.12) | 0.00 | (Ref) | 1.00 | (Ref) |
| **Pneumonia** |  |  |  |  |  |  |  |  |
| SGLT2i | 714 | 170 048 | 4.20 | (3.90 to 4.52) | -2.99 | (-3.49 to -2.49) | 0.58 | (0.53 to 0.64) |
| DPP4i | 1262 | 175 534 | 7.19 | (6.80 to 7.60) | 0.00 | (Ref) | 1.00 | (Ref) |
| **Respiratory failure** |  |  |  |  |  |  |  |  |
| SGLT2i | 16 | 170 560 | 0.09 | (0.06 to 0.15) | -0.17 | (-0.25 to -0.08) | 0.36 | (0.20 to 0.64) |
| DPP4i | 46 | 176 529 | 0.26 | (0.20 to 0.35) | 0.00 | (Ref) | 1.00 | (Ref) |

^*^Users of SGLT2is were propensity-score matched to users of DPP4is in a 1:1 ratio.

^†^Defined as a composite end point of acute pulmonary edema, acute respiratory distress syndrome, pneumonia, or respiratory failure.

ARDS, acute respiratory pulmonary syndrome; CKD, chronic kidney disease; DPP4i, dipeptidyl peptidase-4 inhibitor; SGLT2i, sodium glucose cotransporter 2 inhibitor

**Table S20** Sensitivity analysis that shortened the end of the study period.

|  | **No of events** | **Person-years** | **Incidence rate per 1000 person-years** | | **Rate difference per 1000 person-years (95% CI)** | | **Hazard ratio^*^ (95% CI)** | |
| --- | --- | --- | --- | --- | --- | --- | --- | --- |
|  |  |  |  |  |  |  |  |  |
| **Respiratory events^†^** |  |  |  |  |  |  |  |  |
| SGLT2i (n=159 519) | 860 | 152 740 | 5.63 | (5.27 to 6.02) | -3.72 | (-4.32 to -3.12) | 0.60 | (0.55 to 0.65) |
| DPP4i (n=159 519) | 1525 | 163 078 | 9.35 | (8.89 to 9.83) | 0.00 | (Ref) | 1.00 | (Ref) |
| **Acute pulmonary edema** |  |  |  |  |  |  |  |  |
| SGLT2i | 22 | 153 298 | 0.14 | (0.09 to 0.22) | -0.14 | (-0.24 to -0.04) | 0.50 | (0.30 to 0.82) |
| DPP4i | 47 | 164 111 | 0.29 | (0.22 to 0.38) | 0.00 | (Ref) | 1.00 | (Ref) |
| **ARDS** |  |  |  |  |  |  |  |  |
| SGLT2i | 6 | 153 305 | 0.04 | (0.02 to 0.09) | -0.02 | (-0.06 to 0.03) | 0.70 | (0.25 to 1.96) |
| DPP4i | 9 | 164 136 | 0.05 | (0.03 to 0.11) | 0.00 | (Ref) | 1.00 | (Ref) |
| **Pneumonia** |  |  |  |  |  |  |  |  |
| SGLT2i | 827 | 152 755 | 5.41 | (5.06 to 5.80) | -3.47 | (-4.06 to -2.88) | 0.61 | (0.56 to 0.66) |
| DPP4i | 1449 | 163 114 | 8.88 | (8.44 to 9.35) | 0.00 | (Ref) | 1.00 | (Ref) |
| **Respiratory failure** |  |  |  |  |  |  |  |  |
| SGLT2i | 22 | 153 297 | 0.14 | (0.09 to 0.22) | -0.21 | (-0.32 to -0.10) | 0.40 | (0.25 to 0.66) |
| DPP4i | 58 | 164 114 | 0.35 | (0.27 to 0.46) | 0.00 | (Ref) | 1.00 | (Ref) |

^*^Users of SGLT2is were propensity-score matched to users of DPP4is in a 1:1 ratio.

^†^Defined as a composite end point of acute pulmonary edema, acute respiratory distress syndrome, pneumonia, or respiratory failure.

ARDS, acute respiratory pulmonary syndrome; CKD, chronic kidney disease; DPP4i, dipeptidyl peptidase-4 inhibitor; SGLT2i, sodium glucose cotransporter 2 inhibitor

**Table S21** Association between SGLT2is used as monotherapy and risk of respiratory events.

|  | **No of events** | **Person-years** | **Incidence rate per 1000 person-years** | | **Rate difference per 1000 person-years (95% CI)** | | **Hazard ratio^*^ (95% CI)** | |
| --- | --- | --- | --- | --- | --- | --- | --- | --- |
|  |  |  |  |  |  |  |  |  |
| **Respiratory events^†^** |  |  |  |  |  |  |  |  |
| SGLT2i (n=32 257) | 127 | 19 752 | 6.43 | (5.40 to 7.65) | -3.34 | (-5.14 to -1.54) | 0.66 | (0.53 to 0.83) |
| DPP4i (n=32 257) | 185 | 18 939 | 9.77 | (8.46 to 11.28) | 0.00 | (Ref) | 1.00 | (Ref) |
| **Acute pulmonary edema** |  |  |  |  |  |  |  |  |
| SGLT2i | 5 | 19 827 | 0.25 | (0.10 to 0.61) | -0.12 | (-0.47 to 0.24) | 0.69 | (0.22 to 2.16) |
| DPP4i | 7 | 19 029 | 0.37 | (0.18 to 0.77) | 0.00 | (Ref) | 1.00 | (Ref) |
| **ARDS** |  |  |  |  |  |  |  |  |
| SGLT2i | 3 | 19 828 | 0.15 | (0.05 to 0.47) | 0.10 | (-0.10 to 0.30) | 2.86 | (0.30 to 27.52) |
| DPP4i | 1 | 19 031 | 0.05 | (0.01 to 0.37) | 0.00 | (Ref) | 1.00 | (Ref) |
| **Pneumonia** |  |  |  |  |  |  |  |  |
| SGLT2i | 120 | 19 755 | 6.07 | (5.08 to 7.26) | -3.16 | (-4.91 to -1.42) | 0.66 | (0.52 to 0.83) |
| DPP4i | 175 | 18 941 | 9.24 | (7.97 to 10.71) | 0.00 | (Ref) | 1.00 | (Ref) |
| **Respiratory failure** |  |  |  |  |  |  |  |  |
| SGLT2i | 4 | 19 826 | 0.20 | (0.08 to 0.54) | -0.22 | (-0.57 to 0.13) | 0.49 | (0.15 to 1.63) |
| DPP4i | 8 | 19 030 | 0.42 | (0.21 to 0.84) | 0.00 | (Ref) | 1.00 | (Ref) |

^*^Users of SGLT2is were propensity-score matched to users of DPP4is in a 1:1 ratio.

^†^Defined as a composite end point of acute pulmonary edema, acute respiratory distress syndrome, pneumonia, or respiratory failure.

ARDS, acute respiratory pulmonary syndrome; CKD, chronic kidney disease; DPP4i, dipeptidyl peptidase-4 inhibitor; SGLT2i, sodium glucose cotransporter 2 inhibitor

**Table S22** Association between SGLT2is added on to metformin and risk of respiratory events.

|  | **No of events** | **Person-years** | **Incidence rate per 1000 person-years** | | **Rate difference per 1000 person-years (95% CI)** | | **Hazard ratio^*^ (95% CI)** | |
| --- | --- | --- | --- | --- | --- | --- | --- | --- |
|  |  |  |  |  |  |  |  |  |
| **Respiratory events^†^** |  |  |  |  |  |  |  |  |
| SGLT2i (n=121 332) | 374 | 108 967 | 3.43 | (3.10 to 3.80) | -1.81 | (-2.38 to -1.24) | 0.66 | (0.58 to 0.75) |
| DPP4i (n=121 332) | 520 | 99 266 | 5.24 | (4.81 to 5.71) | 0.00 | (Ref) | 1.00 | (Ref) |
| **Acute pulmonary edema** |  |  |  |  |  |  |  |  |
| SGLT2i | 4 | 109 236 | 0.04 | (0.01 to 0.10) | -0.02 | (-0.08 to 0.04) | 0.62 | (0.18 to 2.21) |
| DPP4i | 6 | 99 622 | 0.06 | (0.03 to 0.13) | 0.00 | (Ref) | 1.00 | (Ref) |
| **ARDS** |  |  |  |  |  |  |  |  |
| SGLT2i | 3 | 109 236 | 0.03 | (0.01 to 0.09) | -0.01 | (-0.06 to 0.04) | 0.68 | (0.15 to 3.04) |
| DPP4i | 4 | 99 624 | 0.04 | (0.02 to 0.11) | 0.00 | (Ref) | 1.00 | (Ref) |
| **Pneumonia** |  |  |  |  |  |  |  |  |
| SGLT2i | 363 | 108 967 | 3.33 | (3.01 to 3.69) | -1.74 | (-2.30 to -1.18) | 0.66 | (0.58 to 0.76) |
| DPP4i | 503 | 99 270 | 5.07 | (4.64 to 5.53) | 0.00 | (Ref) | 1.00 | (Ref) |
| **Respiratory failure** |  |  |  |  |  |  |  |  |
| SGLT2i | 7 | 109 236 | 0.06 | (0.03 to 0.13) | -0.06 | (-0.14 to 0.03) | 0.53 | (0.21 to 1.34) |
| DPP4i | 12 | 99 622 | 0.12 | (0.07 to 0.21) | 0.00 | (Ref) | 1.00 | (Ref) |

^*^Users of SGLT2is were propensity-score matched to users of DPP4is in a 1:1 ratio.

^†^Defined as a composite end point of acute pulmonary edema, acute respiratory distress syndrome, pneumonia, or respiratory failure.

ARDS, acute respiratory pulmonary syndrome; CKD, chronic kidney disease; DPP4i, dipeptidyl peptidase-4 inhibitor; SGLT2i, sodium glucose cotransporter 2 inhibitor

**Table S23** Association between SGLT2is and risk of pneumonia, according to its etiology.

|  | **No of events** | **Person-years** | **Incidence rate per 1000 person-years** | | **Rate difference per 1000 person-years (95% CI)** | | **Hazard ratio^*^ (95% CI)** | |
| --- | --- | --- | --- | --- | --- | --- | --- | --- |
| **Bacterial pneumonia^†^** |  |  |  |  |  |  |  |  |
| SGLT2i | 129 | 226 362 | 0.57 | (0.48 to 0.68) | -0.29 | (-0.44 to -0.13) | 0.66 | (0.53 to 0.82) |
| DPP4i | 207 | 241 573 | 0.86 | (0.75 to 0.98) | 0.00 | (Ref) | 1.00 | (Ref) |
| **Viral pneumonia**^‡^ |  |  |  |  |  |  |  |  |
| SGLT2i | 274 | 226 156 | 1.21 | (1.08 to 1.36) | -0.49 | (-0.71 to -0.27) | 0.70 | (0.60 to 0.82) |
| DPP4i | 410 | 241 225 | 1.70 | (1.54 to 1.87) | 0.00 | (Ref) | 1.00 | (Ref) |
| **Other pneumonia**^¶^ |  |  |  |  |  |  |  |  |
| SGLT2i | 599 | 226 010 | 2.65 | (2.45 to 2.87) | -2.11 | (-2.45 to -1.76) | 0.55 | (0.50 to 0.61) |
| DPP4i | 1145 | 240 758 | 4.76 | (4.49 to 5.04) | 0.00 | (Ref) | 1.00 | (Ref) |

^*^Users of SGLT2is were propensity-score matched to users of DPP4is in a 1:1 ratio.

^†^Defined using the following ICD-10 diagnostic codes: J13, J14, J15

^‡^Defined using the following ICD-10 diagnostic codes: J10, J11, J12, J14, B012, B052, B250

^¶^Defined using the following ICD-10 diagnostic codes: J16, J18

DPP4i, dipeptidyl peptidase-4 inhibitor; SGLT2i, sodium glucose cotransporter 2 inhibitor

**Table S24.** Reasons for censoring in the as-treated analysis.

| **Reason for censoring, n (%)** | **SGLT2i**  **(n=205 534)** | | **DPP4i**  **(n=205 534)** | |
| --- | --- | --- | --- | --- |
| Outcome occurrence | 1025 | 0.50 | 1810 | 0.88 |
| Treatment switch to comparator | 34 686 | 16.88 | 18 282 | 8.89 |
| Treatment discontinuation (30-day grace period) | 85 419 | 41.56 | 97 311 | 47.35 |
| In-hospital death | 405 | 0.20 | 881 | 0.43 |
| End of study period (Dec 31, 2020) | 83 999 | 40.87 | 87 250 | 42.45 |

DPP4i, dipeptidyl peptidase-4 inhibitor; SGLT2i, sodium glucose cotransporter 2 inhibitor
